# Intra-islet glucagon confers β-cell glucose competence for first-phase insulin secretion and favors GLP-1R stimulation by exogenous glucagon

**DOI:** 10.1016/j.jbc.2021.101484

**Published:** 2021-12-09

**Authors:** Over Cabrera, James Ficorilli, Janice Shaw, Felipe Echeverri, Frank Schwede, Oleg G. Chepurny, Colin A. Leech, George G. Holz

**Affiliations:** 1 Lilly Research Laboratories, Eli Lilly and Company, Indianapolis, Indiana, USA; 2 Biorep Technologies, Miami Lakes, Florida, USA; 3 Biolog Life Science Institute GmbH & Co KG, Bremen, Germany; 4 Department of Medicine, State University of New York (SUNY) Upstate Medical University, Syracuse, New York, USA; 5 Department of Pharmacology, State University of New York (SUNY) Upstate Medical University, Syracuse, New York, USA

**Keywords:** glucagon, GLP-1, cAMP, islet, insulin secretion, AUC, area-under-the-curve, BSA, bovine serum albumin, CM, conditioned medium, GcgR, glucagon receptor, GLP-1R, glucagon-like peptide-1 receptor, GRA, GcgR antagonist, GSIS, glucose-stimulated insulin secretion, IVGTT, intravenous glucose tolerance test, OGTT, oral glucose tolerance test, SES, standard extracellular saline, T2D, type 2 diabetes

## Abstract

We report that intra-islet glucagon secreted from α-cells signals through β-cell glucagon and GLP-1 receptors (GcgR and GLP-1R), thereby conferring to rat islets their competence to exhibit first-phase glucose-stimulated insulin secretion (GSIS). Thus, in islets not treated with exogenous glucagon or GLP-1, first-phase GSIS is abolished by a GcgR antagonist (LY2786890) or a GLP-1R antagonist (Ex[9–39]). Mechanistically, glucose competence in response to intra-islet glucagon is conditional on β-cell cAMP signaling because it is blocked by the cAMP antagonist prodrug Rp-8-Br-cAMPS-pAB. In its role as a paracrine hormone, intra-islet glucagon binds with high affinity to the GcgR, while also exerting a “spillover” effect to bind with low affinity to the GLP-1R. This produces a right shift of the concentration-response relationship for the potentiation of GSIS by exogenous glucagon. Thus, 0.3 nM glucagon fails to potentiate GSIS, as expected if similar concentrations of intra-islet glucagon already occupy the GcgR. However, 10 to 30 nM glucagon effectively engages the β-cell GLP-1R to potentiate GSIS, an action blocked by Ex[9–39] but not LY2786890. Finally, we report that the action of intra-islet glucagon to support insulin secretion requires a step-wise increase of glucose concentration to trigger first-phase GSIS. It is not measurable when GSIS is stimulated by a gradient of increasing glucose concentrations, as occurs during an oral glucose tolerance test *in vivo*. Collectively, such findings are understandable if defective intra-islet glucagon action contributes to the characteristic loss of first-phase GSIS in an intravenous glucose tolerance test, that is, diagnostic of type 2 diabetes in the clinical setting.

Pancreatic β-cells located in the islets of Langerhans serve as blood glucose sensors in that they secrete insulin in response to the rise of blood glucose concentration that occurs after a meal (1). This glucose-stimulated insulin secretion (GSIS) requires glucose uptake and oxidative glucose metabolism that generates key ionic (Ca^2+^) and metabolic coupling factors (ATP, glutamate, NADPH, and monoacylglycerol) that act intracellularly to promote exocytosis of insulin packaged within large dense core secretory granules (2). Stimulus-secretion coupling of this type is of high importance to the maintenance of systemic glucose homeostasis by virtue of the fact that circulating insulin promotes glucose uptake while also inhibiting glucose release in target tissues that express insulin receptors (3). Thus, defective GSIS leads to insulin insufficiency that predisposes to hyperglycemia with consequent transition to type 2 diabetes (T2D) (4).

In humans intravenously infused with glucose, it is possible to study the kinetics of GSIS in response to a step-wise increase of blood glucose concentration. This approach reveals two distinct kinetic components of GSIS that are defined as first and second phase insulin secretion. Remarkably, the loss of first-phase GSIS is an accurate predictor of T2D at early stages in the disease process (5, 6, 7). Why this is the case is not known, but an attractive hypothesis is that the “competence” of β-cells to release insulin in response to glucose is conditional on an intra-islet paracrine hormone signaling mechanism. In this scenario, glucagon released from islet α-cells acts at β-cell glucagon receptors (GcgR) and/or glucagon-like peptide-1 receptors (GLP-1R) to stimulate the production of cAMP that acts as a necessary cofactor to support GSIS (8, 9, 10, 11, 12, 13, 14, 15, 16, 17, 18, 19, 20), thereby maintaining “β-cell tone” (15). Thus, a diminished intra-islet paracrine hormone action of glucagon might lead to a loss of β-cell glucose competence manifest as a loss of first and/or second phase GSIS. Intriguingly, this defect might be corrected by β-cell cAMP-elevating agents such as dulaglutide or semaglutide that are commonly prescribed for treatment of T2D (21, 22, 23, 24).

What has not been established to date is whether intra-islet glucagon acts through the GcgR and/or GLP-1R to exert a selective effect to upregulate first and/or second phase GSIS. A selective effect is possible based on our previous report that the cAMP antagonist prodrug Rp-8-Br-cAMPS-pAB abolished first-phase but not second-phase GSIS in response to glucose alone (25). Rp-8-Br-cAMPS-pAB is a highly membrane permeable para-acetoxybenzyl (pAB) ester prodrug that is bioactivated by cytosolic esterases to liberate unconjugated Rp-8-Br-cAMPS that competitively inhibits stimulatory effects of endogenous cAMP at the cAMP-binding domains of cAMP-dependent PKA (25) and cAMP-regulated guanine nucleotide exchange factors Epac1 and Epac2 (25). In this regard, PKA (26, 27, 28, 29, 30, 31) and Epac2 (29, 31, 32, 33, 34, 35, 36, 37, 38, 39) are the principal targets of cAMP relevant to β-cell insulin secretion.

To determine if intra-islet glucagon exerts its secretagogue action primarily at the GcgR, we treated islets with the GcgR antagonist (GRA) monoclonal antibody LY2786890 (*a.k.a.*, Ab-4) (40). Because the volume-restricted microenvironment of islets may allow glucagon to accumulate at concentrations high enough to stimulate the GLP-1R (41), we also tested the GLP-1R antagonist Exendin-[9–39] (Ex[9–39]) (42, 43). Complementary studies using FRET assays to detect cAMP, or immunoassays to detect glucagon, allowed formulation of a new receptor occupancy model of glucagon action that explains the previously reported paradoxical finding that the GLP-1R mediates actions of exogenous glucagon to stimulate insulin secretion (9, 44). Finally, we report that the action of intra-islet glucagon to support insulin secretion requires a step-wise increase of glucose concentration to trigger first-phase GSIS. It is not measurable when GSIS is stimulated by a gradient of increasing glucose concentrations, as occurs during an oral glucose tolerance test (OGTT) *in vivo* (45). Collectively, such findings are understandable if defective intra-islet glucagon action contributes to a characteristic loss of first-phase GSIS in the intravenous glucose tolerance test that is diagnostic of T2D in the clinical setting (5).

## Results

### Differential control of first and second phase GSIS by glucose and GLP-1

Intra-islet hormones may act as endogenous competence factors to enable GSIS in response to glucose alone. Furthermore, their presence in islets may alter the potency and efficacy of glucagon or GLP-1[7-36]amide (GLP-1) when each is administered as a synthetic peptide. To investigate these possibilities, we established methods that allow automated sampling for fast temporal resolution of first and second phase GSIS. Initially, these methods were validated in perifusion assays using GLP-1R agonists and antagonists, thus revealing novel features concerning GLP-1 insulin secretagogue action. Such studies provided a baseline for subsequent assays using GcgR agonists and antagonists so that the pharmacological properties of GLP-1 and glucagon could be compared.

**Figure 1:**
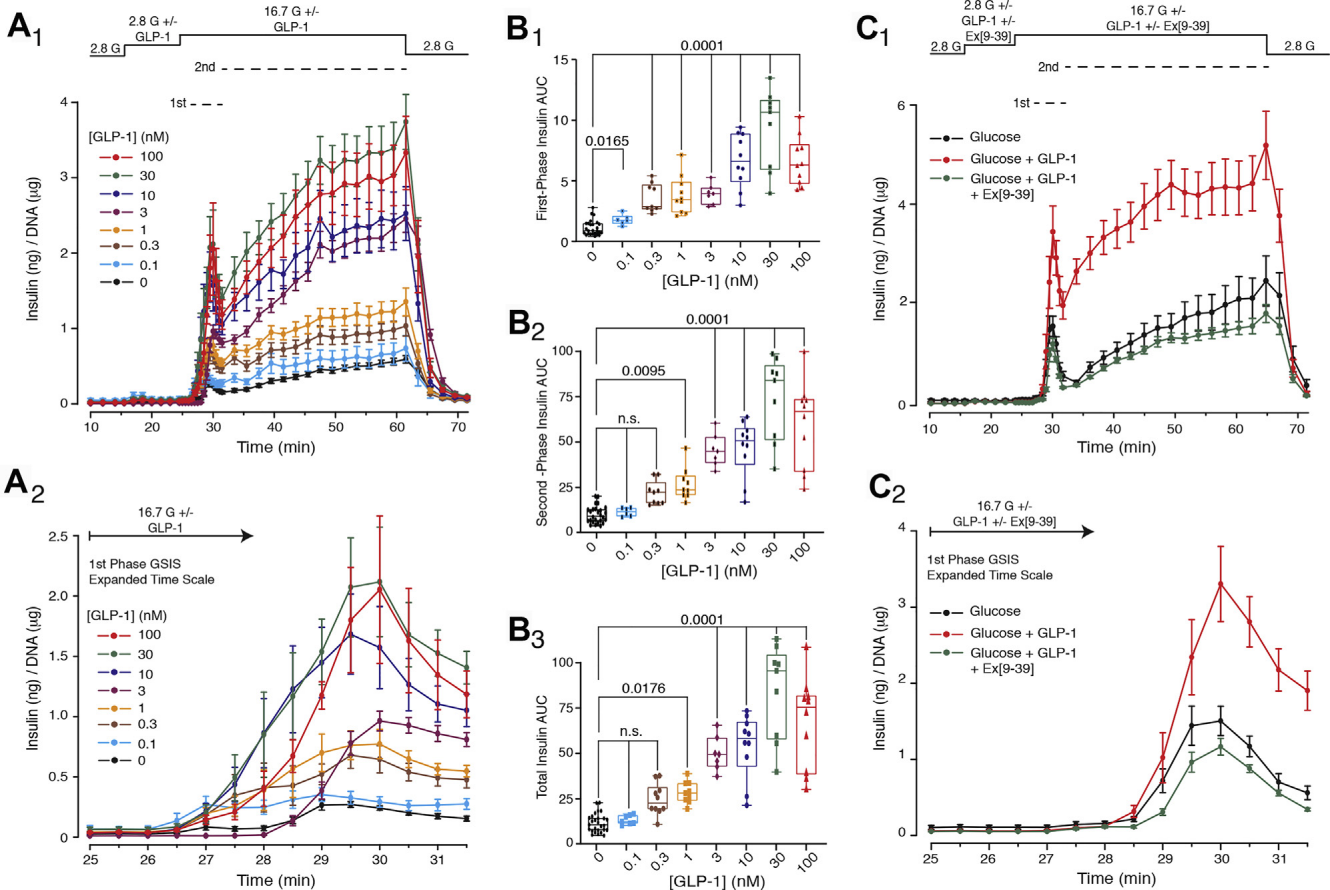
**Concentration-response relationship for potentiation of GSIS by GLP-1 in rat islets.***A*_1_ and *A*_2_, GLP-1 potentiated GSIS that was initiated by a step-wise increase of glucose concentration from 2.8 to 16.7 mM (2.8G and 16.7G). This action of GLP-1 is illustrated on a compressed time scale (*A*_1_) that depicts first and second phase GSIS or on an expanded time scale that depicts first-phase GSIS only (*A*_2_). Individual values are mean ± SEM. The action of GLP-1 was quantified by AUC analysis for first-phase (*B*_1_), second-phase (*B*_2_), and total (*B*_3_; sum of first and second phases) insulin secretion. Note that the statistically significant threshold for GLP-1 agonist action was *ca.* 100 pM when monitoring first-phase GSIS (*B*_1_; *p* value 0.0165), whereas it was 1 nM when monitoring second-phase and total GSIS (*B*_2_ and *B*_3_; *p* values 0.0095 and 0.0176, respectively). The findings in panels *A*_1_–*B*_3_ are averaged data with ANOVA analysis obtained from nine independent experiments. The significance (*p* values) is indicated with accompanying comparisons. Each symbol in the *box* and *whiskers* plots is the AUC value obtained when monitoring GSIS from the islets of a single perifusion chamber. *C*_1_ and *C*_2_, GLP-1R antagonist Ex[9–39] tested at 1 μM blocked the action of 1 nM GLP-1 to potentiate GSIS, as measured in the assays of total GSIS (*C*_1_) or first-phase GSIS (*C*_2_). The findings presented in panels *C*_1_ and *C*_2_ are averaged data from five independent experiments. AUC, area-under-the-curve; n.s., not significant.

Perifusion assays using batch preparations of islets obtained from Sprague–Dawley rats demonstrated the expected potentiation of first and second phase GSIS in response to administered GLP-1 (Fig. 1, *A*_1_ and *A*_2_). This action of GLP-1 was measured under conditions in which the glucose concentration was increased in a step-wise manner from 2.8 to 16.7 mM. Area-under-the-curve (AUC) analysis (Fig. 1, *B*_1_–*B*_3_) revealed that the threshold GLP-1 concentration for potentiation of first-phase GSIS was *ca.* 100 pM (Fig. 1*B*_1_). In contrast, the threshold for potentiation of second-phase GSIS was *ca.* 1 nM (Fig. 1*B*_2_). Thus, we obtained the novel finding that first-phase GSIS was responsive to a tenfold lower concentration of GLP-1 in comparison with second-phase GSIS. As explained in greater detail below, the high potency of administered GLP-1 is expected if levels of intra-islet GLP-1 are so low that there is little or no prior occupancy of the β-cell GLP-1R by endogenous GLP-1. Importantly, the specificity with which GLP-1 exerted its secretagogue action in this assay was established based on the ability of GLP-1R antagonist Ex[9–39] to block the agonist action of GLP-1 (Fig. 1, *C*_1_ and *C*_2_).

**Figure 2:**
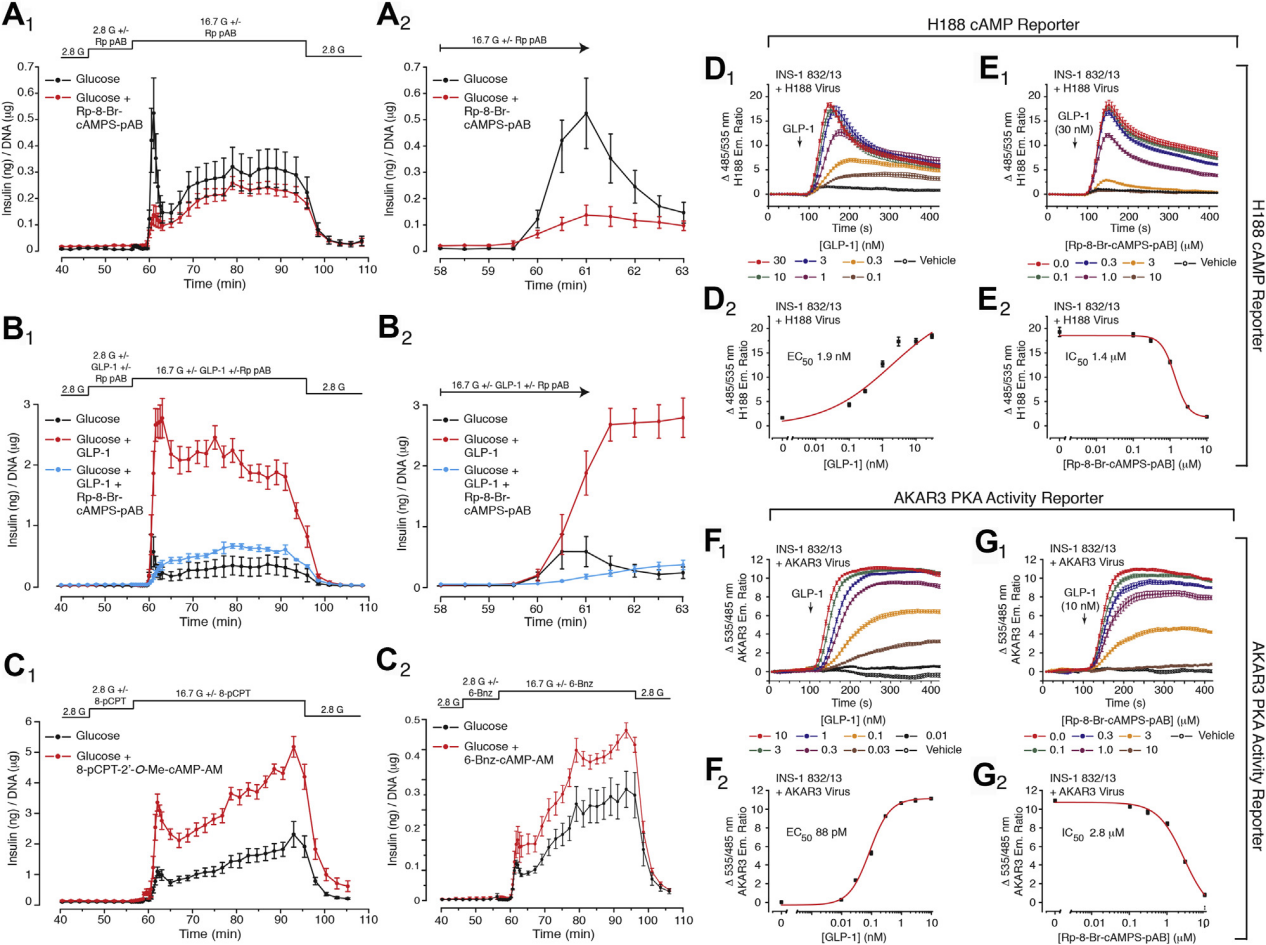
**GLP-1 renders cAMP-dependent exocytosis operational during second-phase GSIS.***A*_1_ and *A*_2_, first but not second phase GSIS stimulated by glucose alone was eliminated during treatment with Rp-8-Br-cAMPS-pAB. Individual values are mean ± SEM. for five independent experiments. *B*_1_ and *B*_2_, GLP-1 potentiated first-phase GSIS, while also potentiating second-phase. Note that both actions of GLP-1 were blocked by Rp-8-Br-cAMPS-pAB. Thus, GLP-1 not only amplified cAMP-dependent exocytosis during first-phase GSIS, but it also recruited cAMP-dependent exocytosis during second-phase. Representative example from seven independent experiments; see Figure 3 for expanded analysis. *C*_1_ and *C*_2_, GSIS was potentiated by the Epac activator 8-pCPT-2′-*O*-Me-cAMP-AM (*C*_1_) or the PKA activator 6-Bnz-cAMP-AM (*C*_2_). Representative examples from six independent experiments. For panels *A*_1_–*C*_2_, the concentrations of Rp-8-Br-cAMPS-pAB, 8-pCPT-2′-*O*-Me-cAMP-AM, and 6-Bnz-cAMP-AM were 10 μM each, whereas for GLP-1, it was 1 nM. *D*_1_ and *D*_2_, FRET assays using INS-1 832/13 cells transduced with H188 demonstrated that GLP-1 raised the levels of cAMP (EC_50_ 1.9 nM). *E*_1_ and *E*_2_, Rp-8-Br-cAMPS-pAB counteracted the stimulatory action of GLP-1 in FRET assays using H188 (IC_50_ 1.4 μM). *F*_1_ and *F*_2_, FRET assays using INS-1 832/13 cells transduced with AKAR3 demonstrated that GLP-1 stimulated PKA activity (EC_50_ 88 pM). *G*_1_ and *G*_2_, Rp-8-Br-cAMPS-pAB counteracted the stimulatory action of GLP-1 in FRET assays using AKAR3 (IC_50_ 2.8 μM).

Next, we confirmed our previous report (25) that the cAMP antagonist prodrug Rp-8-Br-cAMPS-pAB (10 μM) blocked first-phase GSIS in response to glucose alone, while having little ability to reduce second-phase GSIS in response to glucose alone (Fig. 2, *A*_1_ and *A*_2_). We also found that GLP-1 (1 nM) failed to restore first-phase GSIS in rat islets treated with Rp-8-Br-cAMPS-pAB (Fig. 2, *B*_1_ and *B*_2_). Of interest, we obtained the novel finding that GLP-1 enabled cAMP-dependent insulin exocytosis to become operational during second-phase GSIS (Fig. 2*B*_1_). Thus, Rp-8-Br-cAMPS-pAB greatly reduced the amplitude of second-phase GSIS in the presence of GLP-1 (Fig. 2*B*_1_). Note that the residual second-phase GSIS measured during treatment with Rp-8-Br-cAMPS-pAB was of similar magnitude to that measured in the absence of GLP-1. This novel finding reveals that during second-phase GSIS, there is a summation of cAMP-dependent and cAMP-independent processes of insulin exocytosis, each under the control of GLP-1 and glucose, respectively. We also provide evidence that Epac2 and PKA participate in the control of GSIS from rat islets. This concept is consistent with our finding that the first and second phases of GSIS were potentiated by cyclic nucleotide analog prodrugs that are selective activators of Epac (8-pCPT-2′-*O*-Me-cAMP-AM) or PKA (6-Bnz-cAMP-AM) (Fig. 2, *C*_1_ and *C*_2_).

To test if Rp-8-Br-cAMPS-pAB blocks Epac and PKA activation, we used the rat INS-1 832/13 insulin-secreting β-cell line that expresses the endogenous GLP-1R (46). These cells were virally transduced with the FRET reporter H188 that contains the cyclic nucleotide-binding domain of Epac (47). GLP-1 raised the levels of cAMP (EC_50_ 1.9 nM) (Fig. 2, *D*_1_ and *D*_2_), an effect blocked by Rp-8-Br-cAMPS-pAB (IC_50_ 1.4 μM) (Fig. 2, *E*_1_ and *E*_2_). The cells were also transduced with the FRET reporter AKAR3 that is a substrate for PKA and that serves as an indirect readout for binding of cAMP to PKA regulatory subunits (48). GLP-1 stimulated PKA activity (EC_50_ 88 pM) (Fig. 2, *F*_1_ and *F*_2_), an effect blocked by Rp-8-Br-cAMPS-pAB (IC_50_ 2.8 μM). Thus, Epac and PKA activation by cAMP is blocked by Rp-8-Br-cAMPS-pAB.

**Figure 3:**
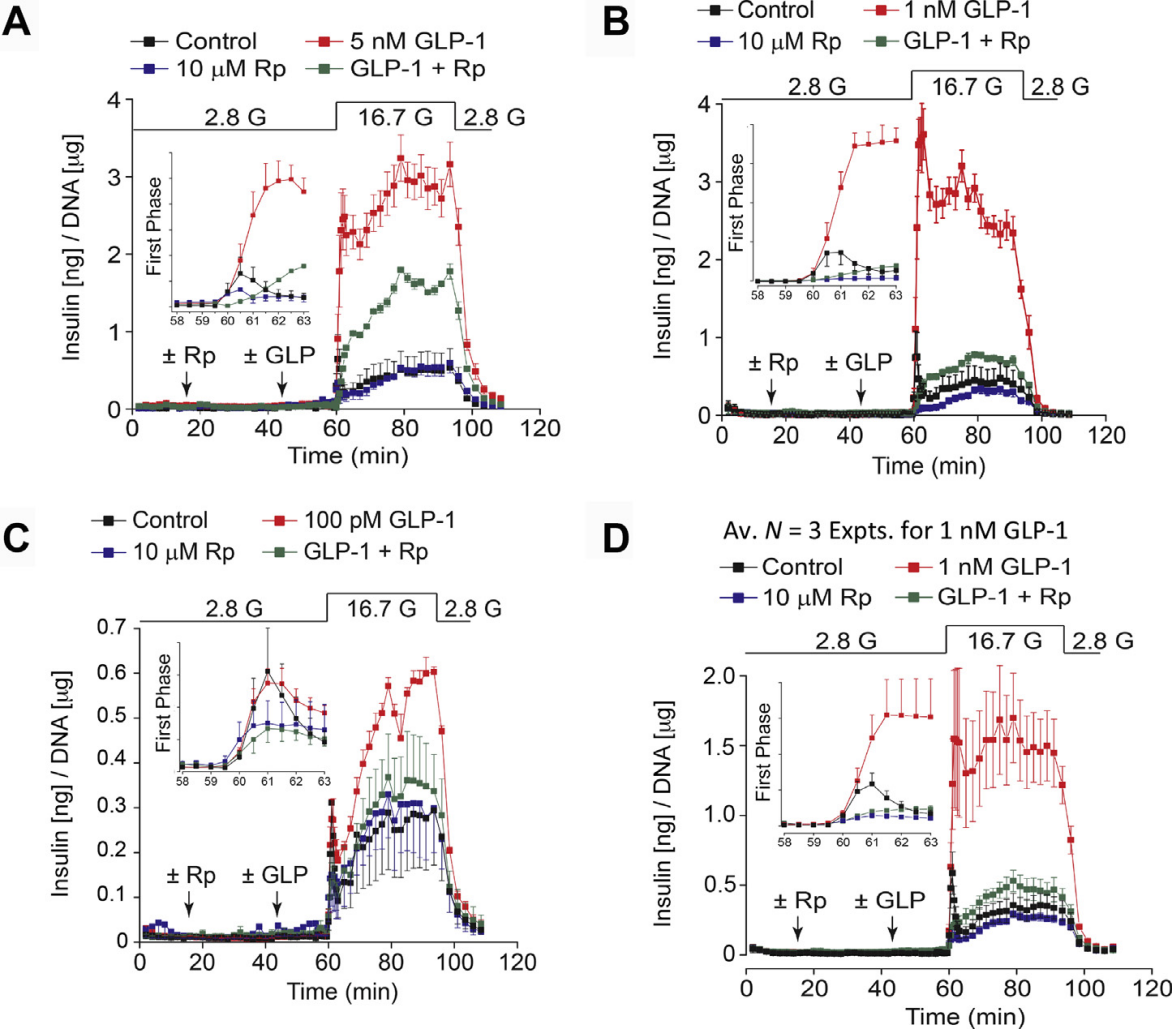
**GLP-1 fails to restore first-phase GSIS in islets treated with Rp-8-Br-cAMPS-pAB.***A*–*C*, Rp-8-Br-cAMPS-pAB (Rp; 10 μM) was tested in assays of GSIS stimulated by glucose alone or glucose combined with GLP-1 (5 and 1 nM, or 100 pM). *D*, illustrated are averaged findings obtained in three independent experiments using 1 nM GLP-1. For each panel, the inset illustrates first-phase GSIS. The individual values are mean ± SEM. Note that Rp-8-Br-cAMPS-pAB strongly suppressed first-phase GSIS in the absence or presence of GLP-1. Also note that GLP-1 recruited cAMP-dependent insulin secretion during second-phase GSIS, whereas in the absence of GLP-1, this kinetic component of GSIS was primarily cAMP-independent. The panels *A*–*C* are representative examples obtained in seven independent experiments.

Having established the suitability of Rp-8-Br-cAMPS-pAB as a tool for evaluation of cAMP signaling in β-cells, a more detailed analysis of its antagonist action was performed across a range of GLP-1 concentrations in assays of GSIS. Consistently, Rp-8-Br-cAMPS-pAB blocked the potentiation of first and second phase GSIS when GLP-1 was tested at concentrations of 5, 1, and 0.1 nM (Fig. 3, *A*–*D*). Interestingly, the antagonist action of Rp-8-Br-cAMPS-pAB was weaker *versus* 5 nM GLP-1 in comparison with 1 nM GLP-1 (*cf.*, Fig. 3, *A* and *B*). This is expected because free Rp-8-Br-cAMPS competes with endogenous cAMP for binding to Epac and PKA, thereby rendering this cAMP analog less effective when concentrations of cAMP rise to high levels in response to 5 nM GLP-1.

Because bioactive GLP-1 circulates at low concentrations, it is of interest that Shigeto *et al.* reported an ability of extremely low concentrations of GLP-1 (0.1–10 pM) to stimulate mouse islet insulin secretion, an effect they explained by PLC and PKC activation (49, 50, 51). In contrast, we obtained the novel finding that concentrations of GLP-1 less than 100 pM failed to stimulate rat islet insulin secretion (Fig. 1, *A*_1_ and *A*_2_), while also failing to stimulate mouse islet insulin secretion using the methods of Shigeto *et al.* (Fig. S1). Using PLC and PKC inhibitors tested by Shigeto *et al.*, we were also unable to confirm PLC- or PKC-mediated actions of GLP-1 to potentiate GSIS from rat islets (Fig. S2). PLC inhibitor U73122 alone potentiated GSIS, an effect not reproduced by the structurally related U73343 that is not a PLC inhibitor, (Fig. S2). Furthermore, GLP-1 retained its ability to potentiate GSIS during treatment with U73122 or U73343 or PKC inhibitors LY 3335531 and Ro 31-8220 (Fig. S2). Still, prior studies demonstrate a novel cAMP signaling mechanism that is stimulated by Epac2 in mouse islets, and that engages PLCε to upregulate PKC activity, IP_3_ production, Ca^2+^ signaling, and insulin secretion (52, 53, 54). Because this nonconventional cAMP signaling is blocked by Rp-8-Br-cAMPS acting at Epac2, it might participate in the control of GSIS by GLP-1, a possibility that remains to be explored in future studies.

### Intra-islet glucagon reduces the potency of administered glucagon in assays of GSIS

**Figure 4:**
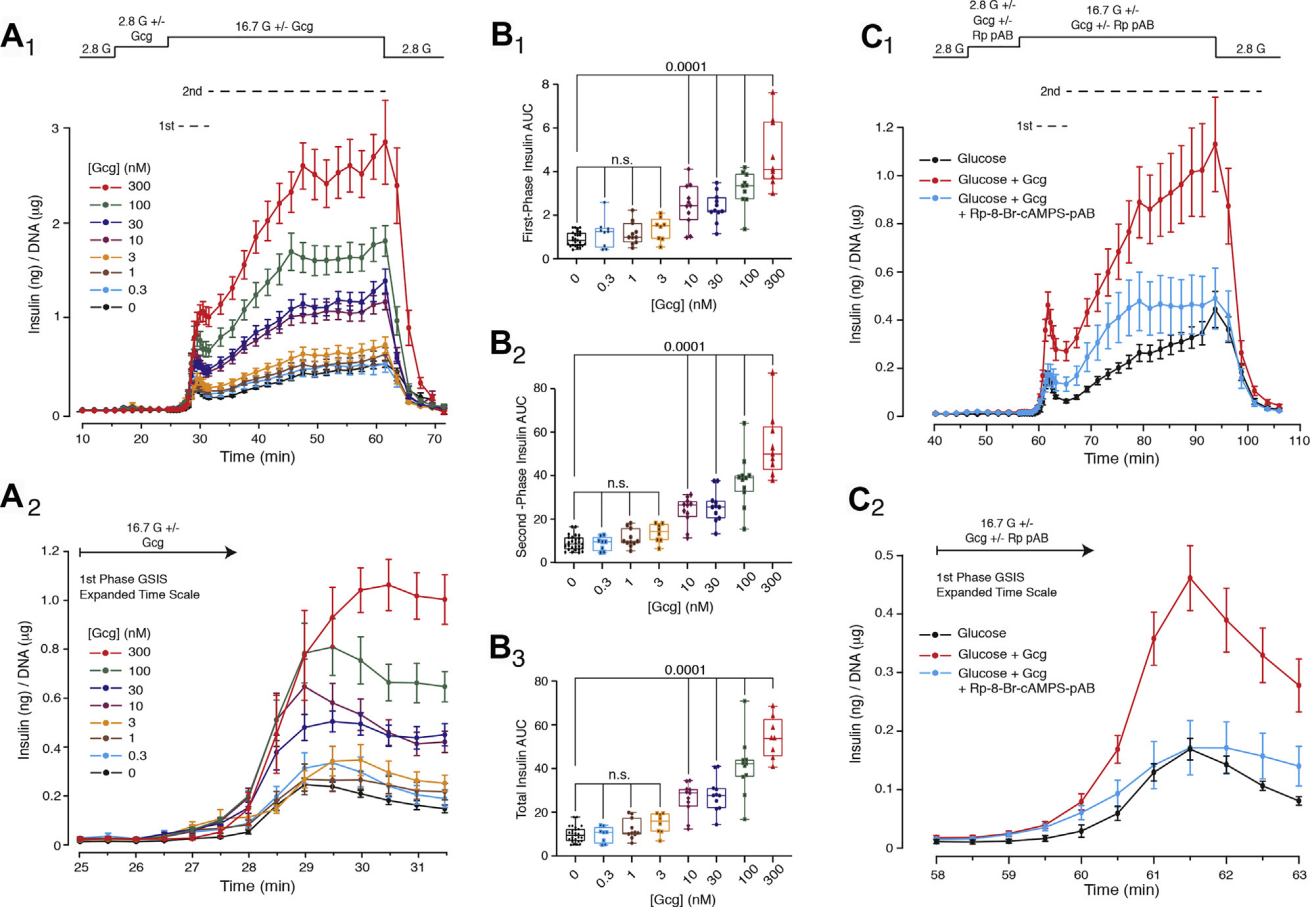
**Concentration-response relationship for potentiation of GSIS by glucagon in rat islets.***A*_1_ and *A*_2_, glucagon potentiated GSIS that was initiated by a step-wise increase of glucose concentration from 2.8 to 16.7 mM. This action of glucagon is illustrated on a compressed time scale (*A*_1_) that depicts first and second phase GSIS or on an expanded time scale that depicts first-phase GSIS only (*A*_2_). The action of glucagon was quantified by AUC analysis in which the concentration-dependent action of glucagon is depicted in *box* and *whiskers* format for first-phase (*B*_1_), second-phase (*B*_2_), and total (*B*_3_) insulin secretion. Note that the statistically significant threshold for glucagon agonist action was *ca.* 10 nM when monitoring first-phase (*B*_1_; *p* value 0.0001), second-phase (*B*_2_, *p* value 0.0001), and total (*B*_3_; *p* value 0.0001) GSIS. The findings in panels *A*_1_–*B*_3_ are averaged data with ANOVA analysis obtained from nine independent experiments. Significance (*p* values) is indicated with accompanying comparisons. Each symbol in the *box* and *whiskers* plots is the AUC value obtained when monitoring GSIS from islets of a single perifusion chamber. *C*_1_ and *C*_2_, Rp-8-Br-cAMPS-pAB tested at 10 μM blocked the potentiation of GSIS by 10 nM glucagon. Representative example from three independent experiments.

The above-summarized pharmacological properties of GLP-1 were compared with those of glucagon in additional assays of GSIS. Unexpectedly, low or intermediate concentrations of glucagon (0.3–3 nM) failed to potentiate GSIS (Fig. 4, *A*_1_ and *A*_2_). AUC analysis revealed that the threshold glucagon concentration for potentiation of first-phase, second-phase, and total GSIS was *ca.* 3 to 10 nM (Fig. 4, *B*_1_–*B*_3_). Still, higher concentrations of glucagon (10–300 nM) exerted a significant effect to potentiate GSIS (Fig. 4, *A*_1_, *A*_2_ and *B*_1_–*B*_3_). The magnitude of this effect of glucagon was comparable to that measured in response to GLP-1 (*cf.*, Fig. 1, *A*_1_ and *A*_2_ *versus* Fig. 4, *A*_1_ and *A*_2_). Furthermore, as for GLP-1, the potentiation of GSIS by glucagon was blocked by Rp-8-Br-cAMPS-pAB (Fig. 4, *C*_1_ and *C*_2_).

**Figure 5:**
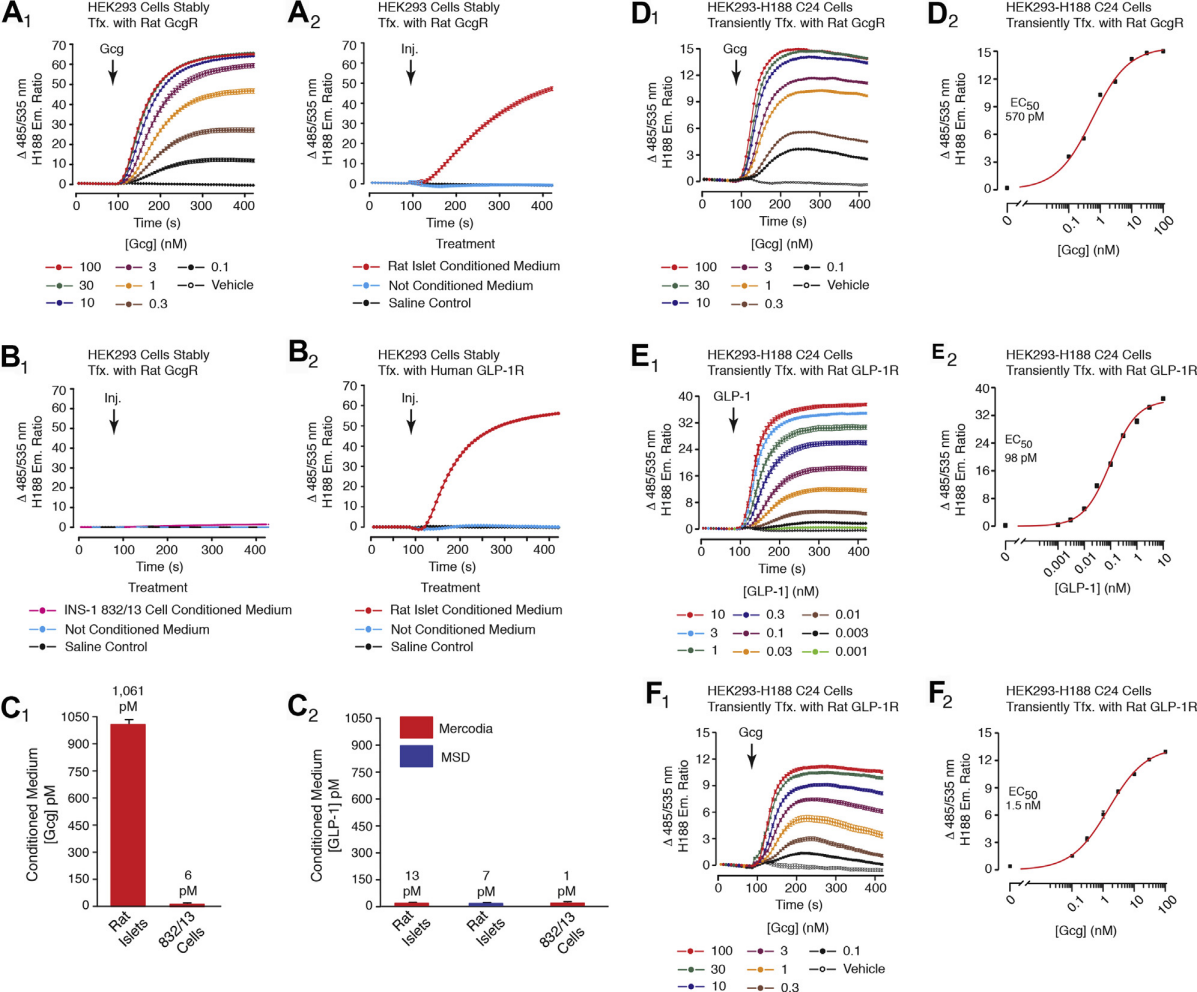
**High levels of glucagon but not GLP-1 are present in conditioned medium of rat islets.***A*_1_, calibration of the glucagon concentration-response relationship in HEK293-GcgR cells transduced with H188. *A*_2_, rat islet conditioned medium increased levels of cAMP in HEK293-GcgR cells, and the magnitude of this change of FRET indicated that the medium contained *ca.* 1 nM glucagon, as inferred from the calibration signal shown in panel *A*_1_. Note that no such signal was measured in medium that was not conditioned. *B*_1_, INS-1 832/13 cell conditioned medium failed to increase levels of cAMP in HEK293-GcgR cells. *B*_2_, rat islet conditioned medium contained GLP-1R agonist activity, most likely because of the presence of glucagon. *C*_1_, ELISA detected 1061 pM glucagon in conditioned medium of rat islets, but only 6 pM in INS-1 832/13 CM. *C*_2_, GLP-1 levels in conditioned medium of rat islets were 13 pM or 7 pM when measured using a Mercodia or MSD ELISA kit, respectively. INS-1 832/13 cell conditioned medium contained 1 pM GLP-1. *D*_1_ and *D*_2_, glucagon increased levels of cAMP in HEK293-GcgR cells (EC_50_ 570 pM). *E*_1_ and *E*_2_, GLP-1 acted to increase levels of cAMP in HEK293-GLP-1R cells (EC_50_ 98 pM). *F*_1_ and *F*_2_, glucagon raised levels of cAMP in HEK293-GLP-1R cells (EC_50_ 1.5 nM).

In comparison with GLP-1, the glucagon concentration-response relationship was right-shifted by *ca.* 30-fold (*cf.*, Fig. 1, *A*_1_ and *A*_2_ *versus* Fig. 4, *A*_1_ and *A*_2_). This is likely to be explained by the presence of intra-islet glucagon which exerts prior occupancy of the GcgR so that stimulatory effects of 0.3 to 3 nM glucagon are not measurable. In fact, conditioned medium (CM) obtained from rat islet cultures (300 islets in 4 ml media and 20 h exposure) contained glucagon that was bioactive in assays of GcgR agonism. This was established in FRET assays using HEK293 cells expressing the GcgR and H188. Under control conditions, glucagon (0.1–100 nM) increased the levels of cAMP, thereby allowing live-cell calibration of its concentration-response relationship (Fig. 5*A*_1_). Based on this calibration, rat islet CM contained *ca.* 1 nM bioactive glucagon (Fig. 5*A*_2_). No effect of CM was measured in HEK293 cells not expressing the GcgR (data not shown). As expected, medium that was not conditioned had no effect (Fig. 5*A*_2_). Furthermore, CM obtained from INS-1 832/13 cell cultures was without GcgR-agonist activity (Fig. 5*B*_1_), as expected for cells that are of β-cell origin. Using ELISA, it was determined that rat islet CM contained 1061 ± 56 pM glucagon, but only 6 pM glucagon for INS-1 832/13 CM (Fig. 5*C*_1_).

We also evaluated the possible presence of GLP-1 in the CM of rat islets. FRET assays using HEK293 cells expressing the GLP-1R demonstrated a stimulatory action of CM to raise levels of cAMP (Fig. 5*B*_2_). However, interpretation of this finding is complicated by the fact that GLP-1 and glucagon both stimulate the GLP-1R. This fact was established in FRET assays that monitor glucagon action at the GcgR (Fig. 5, *D*_1_ and *D*_2_), or GLP-1 action at the GLP-1R (Fig. 5, *E*_1_ and *E*_2_), or glucagon action at the GLP-1R (Fig. 5, *F*_1_ and *F*_2_). Thus, this FRET assay is unable to distinguish between GLP-1 or glucagon in the CM. Therefore, a direct measurement of GLP-1 was performed using ELISA of CM. For rat islets, the CM contained especially low concentrations of GLP-1 (7–13 pM), as was also the case for INS-1 832/13 CM (1 pM) (Fig. 5, *C*_1_ and *C*_2_).

Although it is impossible to measure the true concentration of intra-islet GLP-1, these findings using rat islet CM are significant in that they offer a simple explanation concerning why administered GLP-1, but not glucagon, acts with high potency to potentiate GSIS. Specifically, if intra-islet concentrations of endogenous GLP-1 are low, there will be little prior occupancy of the β-cell GLP-1R when testing administered GLP-1. Still, a “spillover” effect of intra-islet glucagon will exist at the GLP-1R, thereby resulting in partial occupancy of these receptors. Thus, the apparent potency of GLP-1 in assays of GSIS (Fig. 1, *A*_1_, *A*_2_ and *B*_1_–*B*_3_) is less than what is measured when monitoring levels of cAMP in HEK293-H188 GLP-1R cells that do not secrete GLP-1 or glucagon (Fig. 5, *E*_1_ and *E*_2_).

Contrasting with the situation described above for GLP-1, the presence of intra-islet glucagon is expected to result in significant prior occupancy of the β-cell GcgR. Thus, the potency of administered glucagon in assays of GSIS is much less than what is measured when monitoring levels of cAMP in HEK293-H188 GcgR cells (Fig. 5, *D*_1_ and *D*_2_). This phenomenon leads to a right-shift of the concentration-response relationship for glucagon in assays of GSIS, thereby explaining the failure of low concentrations of glucagon to potentiate GSIS (Fig. 4, *A*_1_, *A*_2_ and *B*_1_–*B*_3_).

### First-phase GSIS stimulated by glucose alone is disrupted by GcgR and GLP-1R antagonists

Intra-islet glucagon released from the islet α-cells might serve as a paracrine hormone that enables adjacent β-cells to exhibit first and/or second phase GSIS. This hypothesis was tested in studies of GSIS under conditions in which islets were stimulated with glucose, while also being treated with the GRA monoclonal antibody LY2786890 or the GLP-1R antagonist Ex[9–39]. Accompanying FRET assays monitoring levels of cAMP validated the specificities with which these antagonists acted at the rat GcgR or GLP-1R.

**Figure 6:**
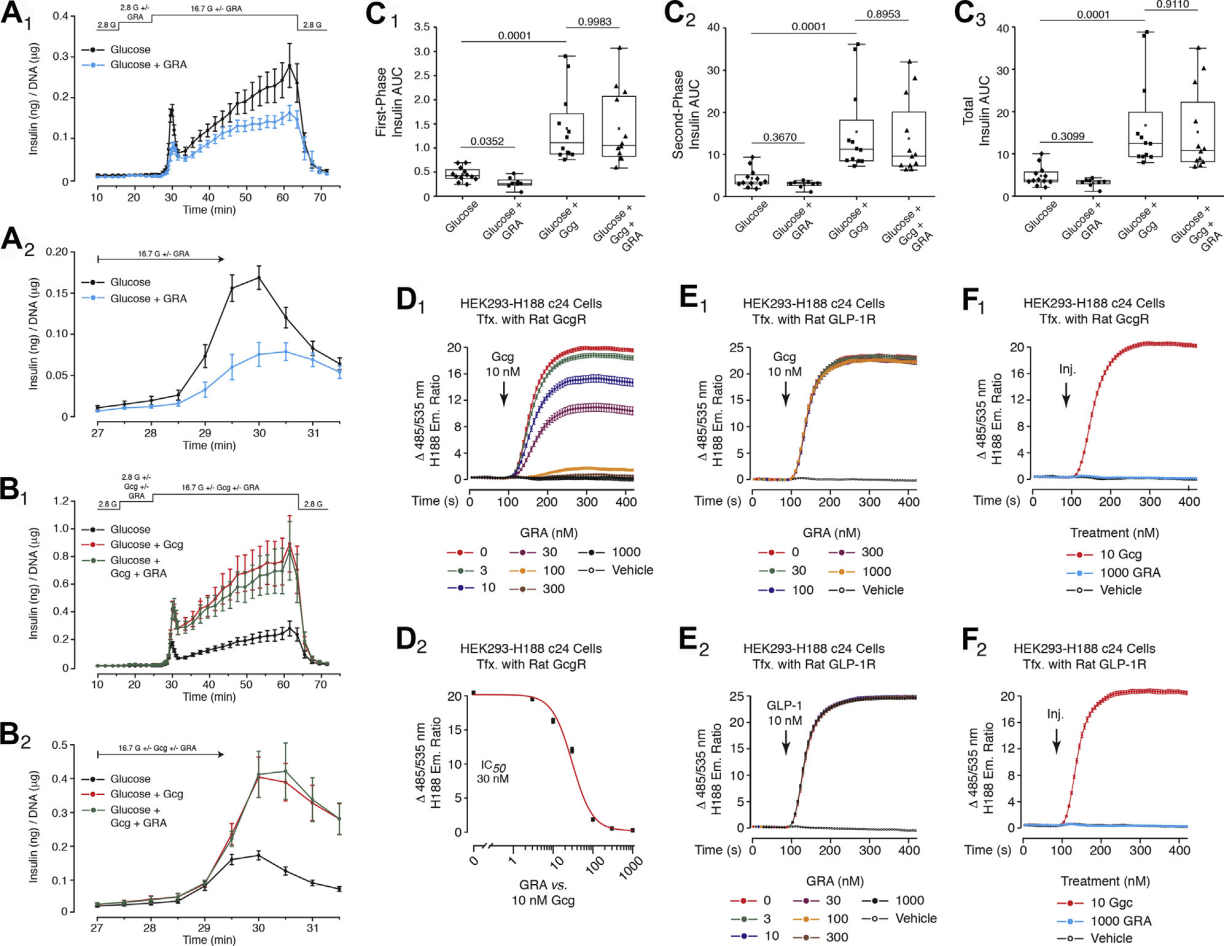
**GRA inhibits first-phase GSIS in response to glucose alone, but is ineffective when glucose is paired with glucagon.***A*_1_ and *A*_2_, treatment of islets with the GRA (70 nM) inhibited first-phase GSIS in response to 16.7 mM glucose alone. A minor inhibitory action of the GRA to inhibit second-phase GSIS was also measurable. *B*_1_ and *B*_2_, the GRA failed to block potentiation of first and second phase GSIS by glucagon (10 nM). *C*_1_–*C*_3_, AUC analysis of GRA antagonist action to alter first-phase, second-phase, or total insulin secretion using experimental designs shown in *A*_1_–*B*_2_. The islets were exposed to 16.7 mM glucose in the presence or absence of glucagon, with or without the GRA. The findings in *A*_1_–*C*_3_ are averaged data analyzed by ANOVA and obtained from four independent identical experiments. Significance (*p* values) is indicated with accompanying comparisons. Each symbol in the *box* and *whiskers* plots is the AUC value obtained when monitoring GSIS from the islets of a single perifusion chamber. *D*_1_ and *D*_2_, target validation of GRA antagonist action (IC_50_ 30 nM) was obtained in FRET assays that monitored levels of cAMP in HEK293-H188 c24 cells transfected with the rat GcgR. The cells were treated with glucagon in the presence or absence of the GRA. *E*_1_ and *E*_2_, the GRA failed to block GLP-1R mediated actions of glucagon or GLP-1 to raise levels of cAMP in HEK293-H188 c24 cells transfected with the rat GLP-1R. *F*_1_ and *F*_2_, the GRA alone failed to alter basal levels of cAMP in HEK293-H188 c24 cells transfected with either the GcgR or GLP-1R.

For islets treated with the GRA (70 nM), a disruption of first-phase GSIS in response to glucose alone was measured, whereas second-phase GSIS was not significantly affected (Fig. 6, *A*_1_ and *A*_2_). Thus, intra-islet glucagon acting at the β-cell GcgR is of critical importance to the generation of first-phase GSIS that is cAMP-dependent (25). In marked contrast, the action of administered glucagon (10 nM) to potentiate first and second phase GSIS was not blocked by the GRA (Fig. 6, *B*_1_ and *B*_2_). These findings are summarized in the accompanying AUC analysis (Fig. 6, *C*_1_–*C*_3_). Thus, intra-islet glucagon engages the β-cell GcgR to enable first-phase insulin secretion in response to glucose alone, whereas a high concentration of administered glucagon acts independent of the GcgR to potentiate GSIS.

GRA antagonist action was specific for the GcgR without any measurable action at the GLP-1R. This was established in FRET assays monitoring levels of cAMP in HEK293-H188 C24 cells stably expressing H188 and transiently transfected with the rat GcgR or GLP-1R. Thus, the GRA (3–1000 nM) blocked the cAMP-elevating action of glucagon (10 nM) at the GcgR (Fig. 6, *D*_1_ and *D*_2_). In contrast, the GRA failed to block glucagon agonist action at the GLP-1R (Fig. 6*E*_1_) or GLP-1 agonist action at the GLP-1R (Fig. 6*E*_2_). Furthermore, the GRA tested alone failed to alter levels of cAMP in cells expressing the GcgR or GLP-1R (Fig. 6, *F*_1_ and *F*_2_). Finally, potentiation of first and second phase GSIS by GLP-1 was not disrupted by the GRA (Fig. S3).

**Figure 7:**
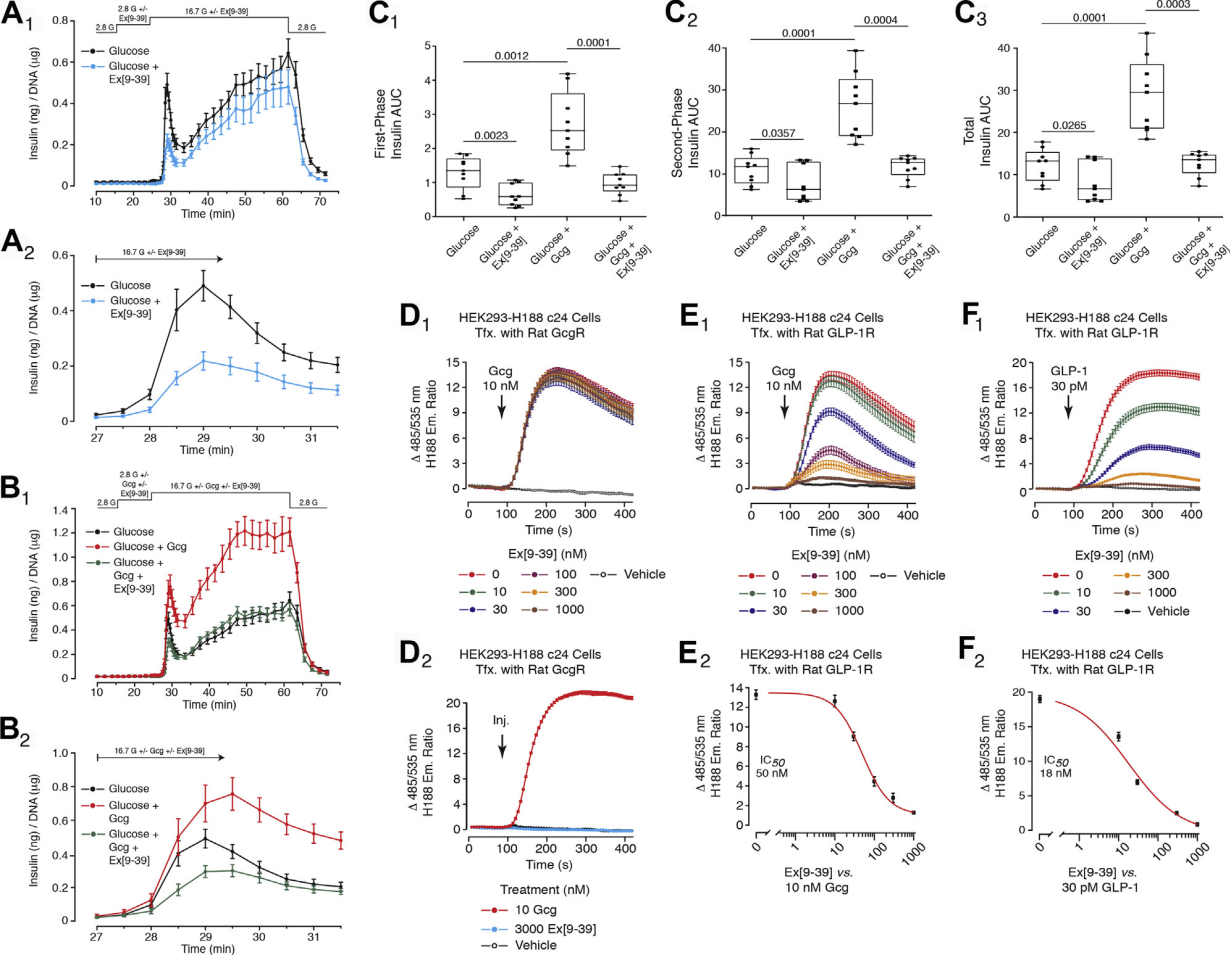
**Ex[9–39] inhibits first-phase GSIS in response to glucose alone, while also blocking the potentiation of GSIS by glucagon.***A*_1_ and *A*_2_, Ex[9–39] (3 μM) inhibited first-phase GSIS in response to 16.7 mM glucose alone. A minor inhibitory action of Ex[9–39] to inhibit second-phase GSIS was also measurable. *B*_1_ and *B*_2_, Ex[9–39] (3 μM) blocked the potentiation of first and second phase GSIS by glucagon (10 nM). *C*_1_–*C*_3_, AUC analysis of Ex[9–39] antagonist action to alter first-phase, second-phase, or total insulin secretion using experimental designs shown in *A*_1_–*B*_2_. The islets were exposed to 16.7 mM glucose in the presence or absence of glucagon, with or without Ex[9–39]. The findings in *A*_1_–*C*_3_ are averaged data analyzed by ANOVA and obtained from three independent identical experiments. Significance (*p* values) is indicated with accompanying comparisons. Each symbol in the *box* and *whiskers* plots is the AUC value obtained when monitoring GSIS from islets of a single perifusion chamber. *D*_1_ and *D*_2_, Ex[9–39] failed to block the action of glucagon to raise levels of cAMP (*D*_1_), while also having no effect on its own (*D*_2_) in HEK293-H188 c24 cells transfected with the rat GcgR. *E*_1_ and *E*_2_, Ex[9–39] blocked the action of glucagon (IC_50_ 50 nM) to raise levels of cAMP in HEK293-H188 c24 cells transfected with the rat GLP-1R. *F*_1_ and *F*_2_, Ex[9–39] blocked the action of GLP-1 (IC_50_ 18 nM) to raise levels of cAMP in HEK293-H188 c24 cells transfected with the rat GLP-1R.

Since first-phase insulin secretion in response to glucose alone is conditional on cAMP signaling, a threshold effect may exist in which levels of β-cell cAMP must exceed some minimal baseline value to support exocytosis. Potentially, intra-islet glucagon acts not only at the GcgR, but also at the GLP-1R to achieve this baseline value. Support for this concept is provided by our finding that first-phase GSIS in response to glucose alone was suppressed by Ex[9–39]. Using methods analogous to those used in studies of the GRA, it was demonstrated that Ex[9–39] (3 μM) suppressed first-phase GSIS in response to glucose, while also exerting a minor inhibitory action to reduce the second-phase GSIS (Fig. 7, *A*_1_ and *A*_2_). Of major significance, Ex[9–39] also blocked the potentiation of GSIS by 10 nM glucagon (Fig. 7, *B*_1_ and *B*_2_). This finding demonstrates that a high concentration of glucagon effectively engages the β-cell GLP-1R. These data are summarized in the accompanying AUC analysis (Fig. 7, *C*_1_–*C*_3_). It is important to note that the findings summarized above are not explained by an off-target action of Ex[9–39] at the GcgR. In HEK293-H188 C24 cells expressing the rat GcgR, the action of glucagon to raise levels of cAMP was not blocked by Ex[9–39] (Fig. 7*D*_1_) nor did Ex[9–39] exert any effect when tested alone (Fig. 7*D*_2_). However, Ex[9–39] blocked the cAMP-elevating actions of glucagon and GLP-1 in HEK293-H188 C24 cells expressing the rat GLP-1R (Fig. 7, *E*_1_ and *E*_2_).

**Figure 8:**
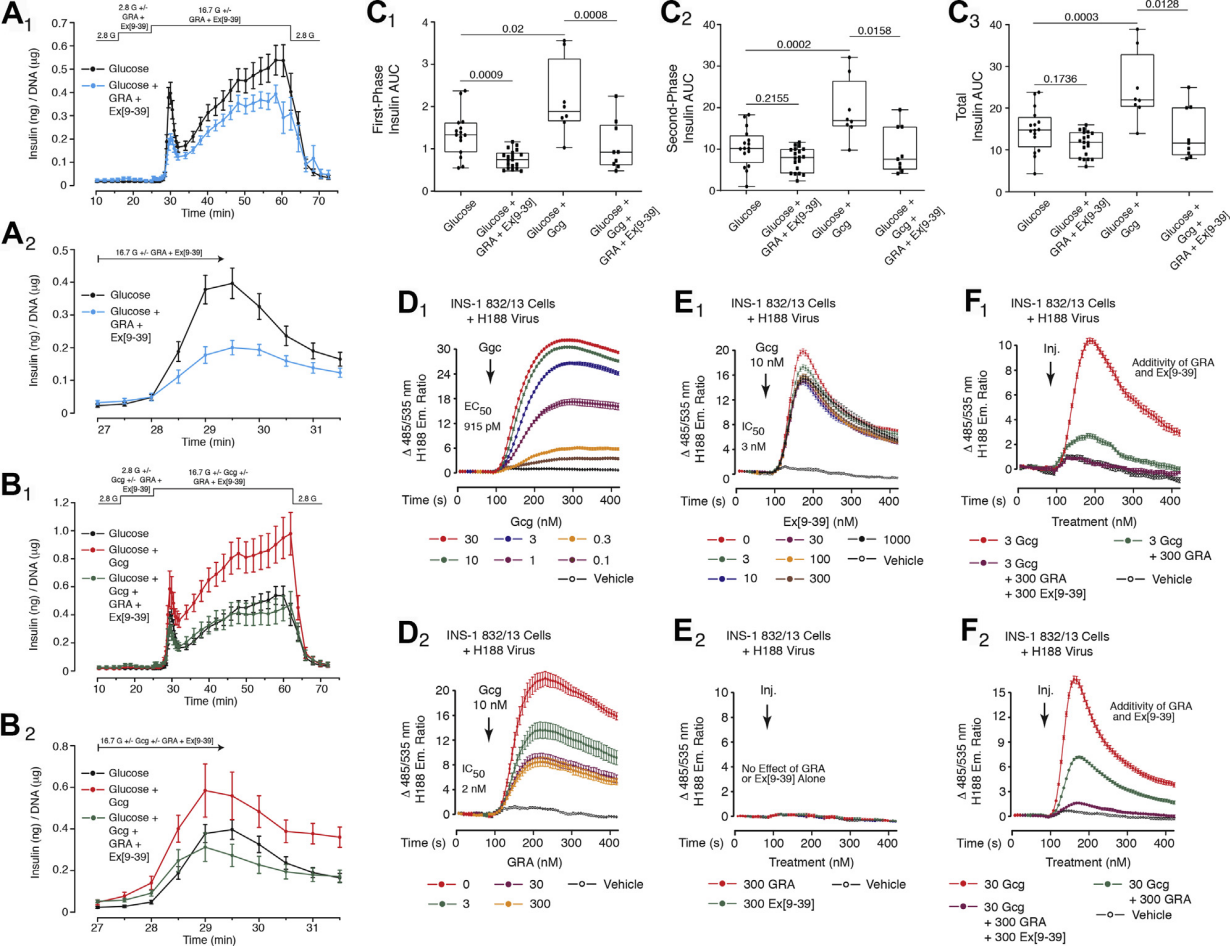
**Assessment of potential additive actions of GRA and Ex[9–39] to inhibit GSIS.***A*_1_ and *A*_2_, combined administration of the GRA (70 nM) and Ex[9–39] (3 μM) inhibited first-phase GSIS in response to glucose alone, but the magnitude of inhibition was similar to that measured when each antagonist was tested alone (*cf.*, Figs. 6 and 7). *B*_1_ and *B*_2_, combined administration of the GRA (70 nM) and Ex[9–39] (3 μM) fully blocked the action of glucagon (10 nM) to potentiate first and second phase GSIS, but the magnitude of inhibition was similar to that measured when each antagonist was tested alone (*cf.*, Figs. 6 and 7). *C*_1_–*C*_3_, AUC analysis summarizing findings obtained for islets treated with the GRA and Ex[9–39] with or without glucagon using experimental designs shown in *A*_1_–*B*_2_. Findings in *A*_1_–*C*_3_ are averaged data analyzed by ANOVA, and obtained from five independent identical experiments. Significance (*p* values) is indicated with accompanying comparisons. Each symbol in the *box* and *whiskers* plots is the AUC value obtained when monitoring GSIS from the islets of a single perifusion chamber. *D*_1_ and *D*_2_, FRET assays demonstrated that glucagon raised levels of cAMP in INS-1 832/13 cells transduced with H188 (EC_50_ 915 pM), an effect not fully blocked by the GRA (IC_50_ 2 nM). *E*_1_ and *E*_2_, Ex[9–39] exerted a weak inhibitory effect to antagonize cAMP-elevating action of 10 nM glucagon, whereas neither Ex[9–39] nor the GRA had effects on their own to alter basal levels of cAMP. *F*_1_ and *F*_2_, GRA and Ex[9–39] exerted an additive effect to block cAMP-elevating actions of 3 or 30 nM glucagon.

A novel prediction derived from this data set is that additive actions of the GRA and Ex[9–39] will not be measurable in assays of first-phase GSIS when these antagonists are tested at saturating concentrations. This prediction is based on the concept that a saturating concentration of each antagonist alone will reduce the levels of cAMP below the minimal threshold value required to allow first-phase GSIS. To test this prediction, the GRA (70 nM) and Ex[9–39] (3 μM) were administered together, while also evaluating the action of glucagon (10 nM) to potentiate GSIS. These experiments demonstrated that dual treatment with the GRA and Ex[9–39] did not reduce first-phase GSIS to a greater extent than what was measured when each was administered alone. Thus, combined treatment with the GRA and Ex[9–39] led to a strong suppression of first-phase GSIS (Fig. 8, *A*_1_ and *A*_2_), but the magnitude of this effect was no greater than what was observed when testing the GRA alone (Fig. 6, *A*_1_ and *A*_2_) or Ex[9–39] alone (Fig. 7, *A*_1_ and *A*_2_). Furthermore, dual treatment with the GRA and Ex[9–39] blocked the potentiation of GSIS by glucagon to the same extent as measured when Ex[9–39] was tested alone (Fig. 8, *B*_1_ and *B*_2_). This finding is expected because Ex[9–39] alone fully blocked the action of glucagon in this assay (Fig. 7, *B*_1_ and *B*_2_). Accompanying AUC analysis summarizes these findings when testing the GRA, Ex[9–39], and glucagon in assays of first-phase, second-phase, and total GSIS (Fig. 8, *C*_1_–*C*_3_).

Although GRA and Ex[9–39] antagonist action is readily studied in HEK293-H188 C24 cells that express the recombinant GcgR and GLP-1R (Figs. 6 and 7), a more physiological approach is to evaluate antagonist action in INS-1 832/13 cells that coexpresses endogenous GcgR and GLP-1R (41). Unlike rat islets, these cells do not secrete significant quantities of glucagon (Fig. 5), and they are especially sensitive to glucagon in FRET assays that monitor levels of cAMP. Thus, glucagon (0.1–30 nM) raised the levels of cAMP in INS-1 832/13 cells, an action reduced but not fully blocked by the GRA (Fig. 8, *D*_1_ and *D*_2_). This is a novel finding in view of the fact that low concentrations of glucagon failed to exert a stimulatory action in assays of rat islet GSIS (Fig. 4). Thus, the concentration of glucagon in the CM of INS-1 832/13 cells (6 pM) is sufficiently low to allow detection of glucagon agonist action at the GcgR when tested at low concentrations (0.1–3 nM). It is also interesting to note that glucagon action in INS-1 832/13 cells was less sensitive to antagonism by Ex[9–39] in comparison with findings obtained in assays of rat islet GSIS (Fig. 8*E*_1_). Importantly, neither the GRA nor Ex[9–39] altered the levels of cAMP on their own (Fig. 8*E*_2_). Finally, a dual agonist action of glucagon was established by demonstrating additive actions of the GRA and Ex[9–39] to suppress cAMP production (Fig. 8, *F*_1_ and *F*_2_).

### GcgR and GLP-1R antagonists fail to reduce GSIS stimulated by a glucose gradient ramp

Oral administration of glucose in an OGTT leads to a slow increase of blood glucose concentration rather than the sudden step-wise increase used to monitor first and second phase GSIS (5, 45). Because this gradual increase of blood glucose levels is a more physiological stimulus for insulin secretion (55), it was of interest to test if intra-islet glucagon regulates GSIS that is measurable *in vitro* using islets exposed to a gradient of slowly increasing concentrations of glucose.

**Figure 9:**
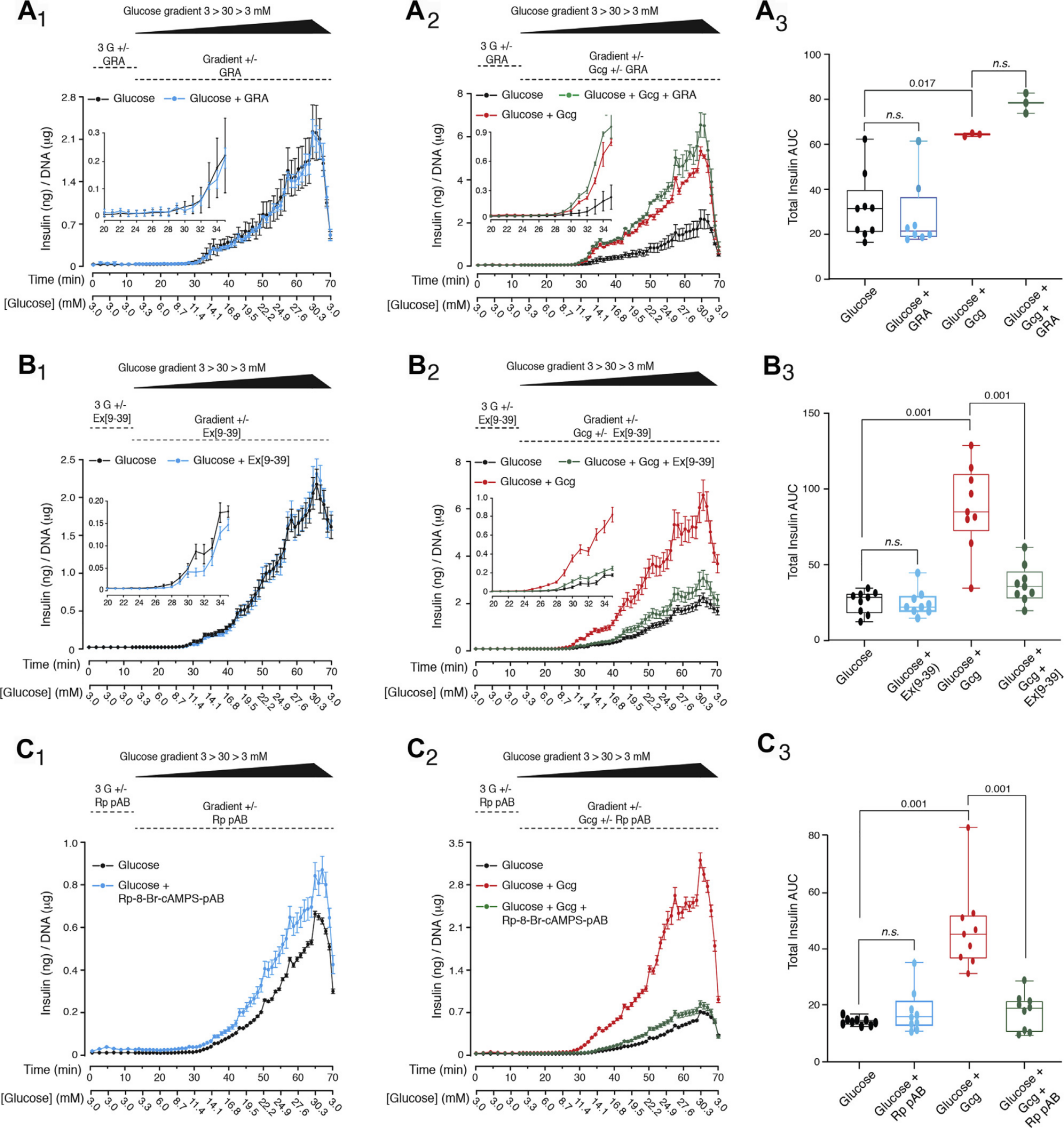
**GRA, Ex[9–39], and Rp-8-Br-cAMPS-pAB fail to inhibit insulin secretion stimulated by a linear gradient of increasing glucose concentrations.***A*_1_–*A*_3_, GRA (70 nM) failed to inhibit GSIS in response to glucose alone (*A*_1_,_3_). Glucagon (10 nM) potentiated GSIS, an effect not inhibited by the GRA (*A*_2_,_3_). These results were obtained in two independent experiments. *B*_1_–*B*_3_, Ex[9–39] (3 μM) failed to inhibit GSIS in response to glucose alone (*B*_1_,_3_). Glucagon (10 nM) potentiated GSIS, an effect inhibited by Ex[9–39] (*B*_2_,_3_). These results were obtained in three independent experiments. *C*_1_–*C*_3_, Rp-8-Br-cAMPS-pAB (10 μM) failed to inhibit GSIS in response to glucose alone, whereas glucagon (10 nM) potentiated GSIS, an effect inhibited by Rp-8-Br-cAMPS-pAB. These results were obtained in three independent experiments. The panels *A*_3_–*C*_3_ illustrate *box* and *whiskers* AUC analyses with accompanying ANOVA derived *p* values where each symbol is the AUC value for the islets of a single perifusion chamber. The islets were perifused under conditions in which the buffer's initial glucose concentration was 3 mM, after which the glucose concentration increased at a rate of 0.27 mM/min, starting at 3.3 mM at *t* = 15 min, and ending at 30.3 mM at *t* = 65 min, after which the glucose concentration was stepped down to 3.0 mM. n.s. not significant; Rp pAB, Rp-8-Br-cAMPS-pAB.

Monophasic insulin secretion from rat islets was measured in response to a linear gradient of glucose concentrations beginning at 3 mM and ending at 30 mM when delivered over a 50 min time course. Unlike its inhibitory effect in step-wise assays of first-phase GSIS, the GRA failed to alter insulin secretion stimulated by a glucose gradient alone (Fig. 9, *A*_1_–*A*_3_). Independent confirmation of this finding was obtained using the GcgR antagonist des-His^1^-Glu^9^-glucagon (Fig. S4). Similarly, Ex[9–39] also failed to alter insulin secretion in response to a glucose gradient alone (Fig. 9, *B*_1_–*B*_3_). Thus, GSIS stimulated by a glucose gradient is not conditional on intra-islet glucagon acting at the GcgR or GLP-1R. Still, glucagon (10 nM) potentiated GSIS in the gradient assay, an action insensitive to the GRA (Fig. 9, *A*_1_–*A*_3_), but blocked by Ex[9–39] (Fig. 9, *B*_1_–*B*_3_). Furthermore, the GRA failed to block the potentiation of insulin secretion by GLP-1 (1 nM), whereas Ex[9–39] was effective (Fig. S5). Thus, glucagon and GLP-1 exerted their stimulatory effects solely through the GLP-1R.

Overall, this pharmacological profile for GcgR and GLP-1R agonist or antagonist action in glucose gradient assays is notable in that it reproduces findings obtained in studies of second-phase GSIS (*cf.*, Figure 6, Figure 7, Figure 8). Potentially, GSIS stimulated by the glucose gradient recruits a mechanism of cAMP-independent insulin secretion that is shared by second-phase GSIS and that is largely insensitive to intra-islet glucagon. In fact, Rp-8-Br-cAMPS-pAB failed to inhibit GSIS measured in the gradient assay, although it blocked the potentiation of GSIS by glucagon (Fig. 9, *C*_1_–*C*_3_).

## Discussion

### Intra-islet glucagon modifies the potency and receptor selectivity of administered glucagon

Here, we applied automated methods of islet perifusion in combination with fast sample rates to monitor the kinetics of insulin secretion so that it would be possible to determine whether intra-islet glucagon acts as a competence factor to support first and/or second phase GSIS. An additional goal was to test a hypothetical receptor occupancy model of GPCR agonist action in which the presence of intra-islet glucagon modifies GcgR and/or GLP-1R agonist potency and selectivity. We also sought to establish the relative importance of cAMP signaling to the genesis of first and second phase GSIS under conditions in which islets were stimulated with glucose alone or glucose in combination with GcgR and GLP-1R agonists. Complementary FRET assays using cAMP biosensors allowed the receptor selectivities of tested compounds to be validated using HEK293 cells expressing recombinant GcgR and GLP-1R or INS-1 832/13 cells that coexpress endogenous GcgR and GLP-1R.

**Figure 10:**
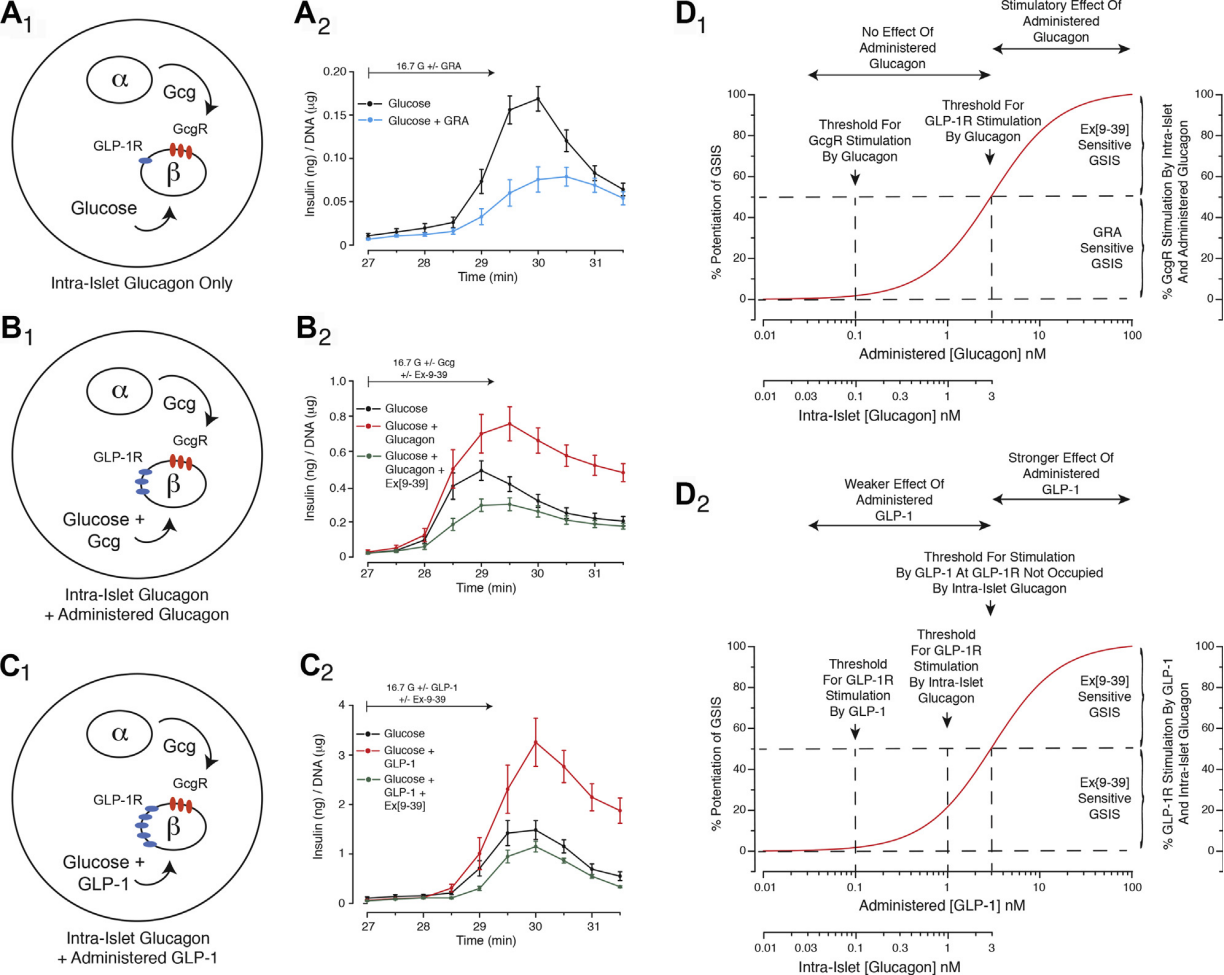
**Summary model.***A*_1_ and *A*_2_, intra-islet glucagon occupies significant numbers of GcgR (*red*), but fewer GLP-1R (*blue*). This intra-islet source of glucagon enables islets to exhibit first-phase GSIS in response to glucose alone, an effect blocked by the GRA. *B*_1_ and *B*_2_, when high concentrations of glucagon are administered in combination with glucose, resultant partial occupancy of the GLP-1R leads to a potentiation of first-phase GSIS, an effect blocked by Ex[9–39]. *C*_1_ and *C*_2_, when high concentrations of GLP-1 are administered in combination with glucose, there is full GLP-1R occupancy, thereby potentiating first-phase GSIS, an effect blocked by Ex[9–39]. *D*_1_, low concentrations of intra-islet glucagon occlude the stimulatory effects of low concentrations of administered glucagon in assays of GSIS. *D*_2_, high concentrations of glucagon engage the GLP-1R to potentiate GSIS. Note that GRA blocks the action of low concentrations of intra-islet glucagon at the β-cell GcgR, whereas Ex[9–39] blocks the action of high concentrations of administered glucagon at the β-cell GLP-1R.

As depicted in the summary model (Fig. 10), these approaches revealed that first-phase GSIS was conditional on a paracrine hormone action of intra-islet glucagon. Although it was not technically possible to measure true intra-islet levels of glucagon, it was instead possible to measure glucagon in the CM of islet cultures. This approach allowed an estimation of the relative concentrations of glucagon and GLP-1 that might exist within the islet interstitium. Based on EC_50_ values for glucagon and GLP-1 obtained in FRET assays that detect cAMP, it is now possible to construct a hypothetical receptor occupancy model (Fig. 10) that seeks to explain how intra-islet glucagon alters the actions of administered glucagon and GLP-1 when each is tested in assays of GSIS. As validated in our FRET assays using H188 and AKAR3, this model assumes that glucagon binds with high affinity to the GcgR but low affinity to the GLP-1R. It also assumes high affinity binding of GLP-1 to the GLP-1R, with no binding to the GcgR.

When neither glucagon nor GLP-1 is administered (Fig. 10*A*_1_), intra-islet glucagon occupies a higher proportion of GcgR (red) in comparison with GLP-1R (blue). Thus, the GRA blocks first-phase GSIS in response to glucose alone (Fig. 10*A*_2_). When intra-islet glucagon is paired with a high concentration of administered glucagon (Fig. 10*B*_1_), additional GLP-1R are recruited so that GLP-1R-stimulated GSIS predominates. Thus, first-phase GSIS is not sensitive to the GRA and is instead blocked by Ex[9–39] (Fig. 10*B*_2_). Finally, when intra-islet glucagon is paired with a saturating concentration of administered GLP-1 (Fig. 10*C*_1_), there is full occupancy of the GLP-1R, and under these conditions Ex[9–39] but not the GRA blocks first-phase GSIS (Fig. 10*C*_2_).

One additional prediction of this model is that prior occupancy of the GcgR by intra-islet glucagon explains why low concentrations (0.1–3 nM) of administered glucagon are ineffective in assays of GSIS. In fact, the assays reported here used islets preequilibrated in 2.8 mM glucose, a concentration that stimulates α-cell glucagon secretion (Fig. S6). This intra-islet endogenous glucagon competes with administered exogenous glucagon for the GcgR, and if concentrations of both are similar, it is not possible to measure a stimulatory action of administered glucagon (Fig. 10*D*_1_). When the concentration of administered glucagon is increased to 10 nM, glucagon achieves its capacity to signal through the GLP-1R (Fig. 10*D*_1_). The net effect is a major right-shift of the glucagon concentration-response relationship when comparing its ability to stimulate insulin secretion from islets, *versus* its ability to stimulate cAMP production in HEK293 GcgR and GLP-1R cell lines that do not secrete glucagon. Note that an especially low concentration of intra-islet GLP-1, as inferred from ELISA of islet CM, will not lead to a major right shift of the GLP-1 concentration-response relationship (Fig. 10*D*_2_). Thus, the threshold for potentiation of GSIS by administered GLP-1 is *ca.* 100 pM, a value closer to that measured in FRET assays using HEK293-GLP-1R cells that do not secrete GLP-1.

### Does intra-islet GLP-1 contribute to β-cell glucose competence?

The concept of β-cell glucose competence was first advanced based on the abilities of glucagon or GLP-1 to restore the capacity of rat β-cells to respond to glucose under conditions of primary cell culture. This restorative effect was measurable in assays of insulin secretion (56, 57, 58), and in patch clamp assays that monitor the ability of glucose to promote the closure of K_ATP_ channels (59, 60). Thus, the question arises as to whether intra-islet GLP-1 serves as a paracrine hormone important to β-cell glucose competence. α-cells synthesize proglucagon that is the precursor for glucagon and possibly GLP-1 under conditions of stress (61, 62, 63, 64, 65, 66). Yet for rat islets, we found no evidence for significant levels of GLP-1, a finding consistent with prior reports using human, mouse, and pig islets (67, 68). This was demonstrated by the immunoassay of rat islet CM using anti-GLP-1 antibodies that detect total GLP-1 measured as fully mature GLP-1[7–36]amide, partially mature GLP-1[7–37], the degradation product GLP-1[9–36]amide, and the precursors GLP-1[1–36]amide and GLP-1[1–37]. The levels of total GLP-1 measured in this manner ranged from 7 to 13 pM, concentrations that are too low to stimulate insulin secretion from rat islets. Furthermore, we were unable to substantiate reports that extremely low concentrations of GLP-1 (0.1–10 pM) stimulate insulin secretion from mouse islets (49, 50, 51). In contrast, the levels of glucagon in rat islet CM were high (1 nM), thereby indicating that glucagon is the primary intra-islet hormone important to β-cell glucose competence under conditions of islet isolation. Interestingly, we obtained the novel finding that Prodo PIM(S) is useful for stabilization of glucagon so that it can be measured after freeze-thawing, while also retaining its agonist activity at the GcgR. This is noteworthy in view of the fact that Prodo PIM(S) is the optimal medium for primary culture of human islets (69). Thus, glucagon secreted from human islets may accumulate in culture medium in its fully bioactive form, thereby allowing it to enhance β-cell functionality.

### Glucagon and GLP-1 enable cAMP-dependent exocytosis during second-phase GSIS

Studies reported here are novel in that they expand upon our prior finding that first-phase but not second-phase insulin secretion in response to glucose alone is blocked by Rp-8-Br-cAMPS-pAB (25). We find that first-phase GSIS is entirely cAMP-dependent, and that this cAMP is generated by intra-islet glucagon acting with high affinity at the β-cell GcgR. A low affinity spillover effect of glucagon to stimulate the GLP-1R is also measurable. Because second-phase GSIS is relatively resistant to Rp-8-Br-cAMPS-pAB, we propose that β-cell cAMP production in response to intra-islet glucagon is not sufficiently strong to upregulate cAMP-dependent exocytosis during second-phase GSIS. In contrast, there is a strong upregulation of second-phase GSIS that is Rp-8-Br-cAMPS-pAB sensitive when the islets are treated with exogenous glucagon or GLP-1. These novel findings indicate that high levels of exogenous glucagon engage the GLP-1R to strongly stimulate cAMP production and to allow cAMP-dependent exocytosis to become operational during second-phase GSIS.

Identifying the mechanism for this switch from cAMP-independent to cAMP-dependent insulin secretion is of great interest. Shibasaki *et al.* (33) reported a cAMP-regulated mechanism of insulin secretion that results from “restless newcomer” exocytosis in which Epac2 activation mobilizes cytoplasmic secretory granules so that they quickly transit to the plasma membrane for release without pausing in a “docked” state. When islets are stimulated with glucose alone, this newcomer exocytosis is more prominent during first-phase as compared with second-phase GSIS. Raising levels of cAMP by treatment with forskolin potentiates newcomer exocytosis during first-phase, while also greatly increasing newcomer exocytosis during second-phase (33). Based on current concepts of cytosolic cAMP “microdomains” (70), we propose the novel hypothesis that first but not second phase newcomer exocytosis is stimulated by spatially restricted cAMP generated by intra-islet glucagon acting at the GcgR. In contrast, exogenous glucagon, acting through the GLP-1R, expands these microdomains to recruit additional newcomer exocytosis during second-phase GSIS.

### Contrasting roles for intra-islet glucagon in assays of monophasic *versus* biphasic GSIS

An additional novel discovery reported here is that GcgR and GLP-1R antagonists fail to inhibit insulin secretion measured in response to an imposed linear increase of the glucose concentration. We interpret this finding to indicate that monophasic insulin secretion measured in the glucose gradient assay is not under the control of intra-islet glucagon. This concept is supported by our finding that monophasic insulin secretion is insensitive to the cAMP antagonist Rp-8-Br-cAMPS-pAB. Still, monophasic insulin secretion is strongly potentiated by glucagon, an action blocked by GLP-1R antagonist Ex[9–39] but not the GcgR antagonists LY2786890 or des-His^1^-Glu^9^-glucagon. Thus, monophasic insulin secretion in response to glucose alone, or in response to glucose paired with glucagon, exhibits pharmacological features similar to second-phase GSIS measured in step-wise assays of biphasic insulin secretion. These surprising findings suggest that under conditions in which glucose is administered by itself, monophasic and second-phase insulin secretion share a common mechanism of cAMP-independent insulin exocytosis that is not contingent on intra-islet glucagon acting at the GcgR or GLP-1R. Instead, our studies using the step-wise assay reveal that the action of intra-islet glucagon is specific for the first-phase GSIS that is cAMP-dependent. This discovery is made possible by our use of rat islets as the test system. Unlike mouse islets that were used in prior studies of intra-islet glucagon action (15, 16), rat islets exhibit substantial second-phase GSIS that is easily distinguishable from first-phase on the basis of kinetics (71).

Because a gradual rather than step-wise increase of blood glucose is the true stimulus for insulin secretion under conditions of oral glucose administration, it might be that intra-islet glucagon plays a less significant role as a determinate of outcomes obtained in an OGTT. Instead, a more important role for intra-islet glucagon is expected when evaluating outcomes obtained in the intravenous glucose tolerance test since infusion of glucose leads to a rapid increase of blood glucose levels and first-phase GSIS.

### Potential physiological relevance

An important question concerns whether nonphysiological accumulation of glucagon within the interstitial space of isolated islets leads to an “apparent” paracrine hormone effect that does not occur *in vivo*. In fact, microvascular blood flow through the islets may efficiently clear glucagon from the interstitial space. If so, our receptor occupancy model (Fig. 10) predicts that GcgR and GLP-1R antagonists will fail to inhibit insulin secretion *in vivo* in response to glucose alone. This prediction is consistent with the findings of Moens *et al.* (11) using a rat perfused pancreas model in which perfusion emulates the effect of blood flow to clear glucagon from the islet's interstitial space. In fact, Moens *et al.* (11) reported that insulin secretion in response to glucose alone was not reduced by GcgR antagonist des-His^1^-Glu^9^-glucagon or GLP-1R antagonist Ex[9–39]. In marked contrast, Svendsen *et al.* (14) used a perfused mouse pancreas model in which Ex[9–39] reduced insulin secretion in response to glucose alone, although a GcgR antagonist was not tested. The reason for this discrepancy is unknown but might reflect a species difference.

Our receptor occupancy model predicts that low concentrations of administered glucagon will act *in vivo* at the β-cell GcgR to stimulate insulin secretion provided that intra-islet glucagon is already cleared from the interstitial space. In fact, Moens *et al.* (11) reported that for perfused rat pancreas, 1 nM glucagon stimulated insulin secretion. Similarly, in studies of perfused mouse pancreas, Svendsen *et al.* (14) reported stimulation of insulin secretion by 0.1 to 10 nM glucagon, an effect mediated by both the GcgR and GLP-1R. Additional studies using genetically engineered mice provide support for *in vivo* paracrine actions of glucagon that are mediated by the GcgR and/or GLP-1R (15, 16). However, compensatory changes of hormone and receptor gene expression in these model systems may cloud the interpretation of findings (72, 73). Thus, the relevance of glucagon acting as an intra-islet paracrine hormone at the GcgR and/or GLP-1R *in vivo* has yet to be fully validated.

### Advantages and limitations of this study

The interpretation of findings reported here for intra-islet control of GSIS by glucagon is based on our use of LY2786890, a monoclonal antibody GcgR antagonist that does not exhibit partial agonist or inverse agonist actions in assays of cAMP, and that does not exert an off-target action at the GLP-1R (41). Although GcgR antagonist MK0893 was recently reported to inhibit glucagon-stimulated insulin secretion from mouse islets (74), it is an allosteric inhibitor that not only antagonizes glucagon action at the GcgR but also the GLP-1R (41). Thus, our studies using LY2786890 and isolated islets more clearly substantiate an ability of intra-islet glucagon to confer β-cell glucose competence for first-phase GSIS in a GcgR-mediate manner. Finally, controversy exists concerning whether Ex[9–39] acts as a pure antagonist or instead as an inverse agonist at the GLP-1R (14, 43, 75). If Ex[9–39] possesses such inverse agonist properties, it may directly block first-phase insulin secretion in a manner that is independent of any action of intra-islet glucagon at the GLP-1R.

## Conclusion

Prior efforts in T2D therapeutics focused on achieving functional restoration of first and second phase GSIS by administered GLP-1R agonists such as exenatide (76). The findings presented here provide a new mechanistic explanation for this beneficial effect. GLP-1R stimulation by GLP-1 leads not simply to a potentiation of first-phase GSIS, but also a recruitment of cAMP-dependent second-phase GSIS that is missing in the absence of GLP-1. Remarkably, high concentrations of glucagon engage the GLP-1R to achieve a similar effect. Increasingly, it is appreciated that glucagon acting at the GcgR and GLP-1R plays an important role in systemic glucose homeostasis (77). Collectively, we expect that new findings reported here will advance T2D drug discovery in that they emphasize the likely clinical relevance of synthetic dual agonist peptides that simultaneously stimulate the GcgR and GLP-1R (78).

## Experimental procedures

### Cell culture

INS-1 832/13 cells were a gift from C. Newgard and were grown in RPMI-1640 culture medium for passaging using the original protocol of Hohmeier *et al.* (46). The parental HEK293 cell line was obtained from the American Type Culture Collection. HEK293 cells stably expressing the rat GcgR (Fig. 5, *A*_1_, *A*_2_ and *B*_1_) at a density of *ca.* 250,000 receptors/cell were obtained from T.P. Sakmar (79). HEK293 cells stably expressing the human GLP-1R (Fig. 5*B*_2_) at a density of *ca.* 150,000 receptors/cell were obtained from Novo Nordisk A/S (80). HEK239-H188 c24 cells stably expressing H188 (Figs. 5, *D*_1_–*F*_2_, 6, *D*_1_–*F*_2_ and 8, *D*_1_–*F*_2_) were generated by O.G. Chepurny in the Holz laboratory (81). All HEK293 cell cultures were maintained in DMEM containing 25 mM glucose and supplemented with 10% fetal bovine serum and 1% penicillin-streptomycin. Cell cultures equilibrated at 37 °C in a humidified incubator that was gassed with 5% CO_2_ were passaged once a week. Culture media and additives were obtained from Thermo Fisher Scientific.

### Cell transfection

HEK293-H188 c24 cells stably expressing H188 were obtained by G418 antibiotic resistance selection using our published methods (81). Transient transfections of HEK293-H188 c24 cells for the expression of GPCRs were performed with Lipofectamine and Plus Reagent (Thermo Fisher) using our published methods (81). Plasmids containing the rat GcgR cDNA (79) or human GLP-1R cDNA (82) were provided by T.P. Sakmar and M. Beinborn, respectively. Adenoviruses for transduction of HEK293 cells were generated by a commercial vendor (ViraQuest) using the shuttle vector pVQAd CMV K-NpA and the H188 plasmid provided by Kees Jalink (47) or the AKAR3 plasmid provided by Jin Zhang (48).

### FRET reporter assay in a 96-well format

HEK293 cells stably expressing recombinant GPCRs were plated at 80% confluence on 96-well clear-bottom assay plates (Costar 3904, Corning) coated with rat tail collagen (Collaborative Biomedical Products). The cells were then transduced for 16 h with H188 virus at a density of *ca.* 60,000 cells/well under conditions in which the multiplicity of infection was equivalent to 25 viral particles per cell. The culture media was removed and replaced by 200 μl/well of a standard extracellular saline (SES) solution supplemented with 11 mM glucose and 0.1% bovine serum albumin. The composition of the SES was (in mM): 138 NaCl, 5.6 KCl, 2.6 CaCl_2_, 1.2 MgCl_2_, 11.1 glucose, and 10 Hepes (295 mOsmol, pH 7.4). Real-time kinetic assays of FRET were performed using a FlexStation 3 microplate reader equipped with excitation and emission light monochromators (Molecular Devices) (41). Excitation light was delivered at 435/9 nm (455 nm cut-off), and emitted light was detected at 485/15 nm (CFP) or 535/15 nm (YFP). The emission intensities were the averages of 12 excitation flashes for each time point per well. Test solutions dissolved in SES were placed in V-bottom 96-well plates (Greiner Bio-One), and an automated pipetting procedure was used to transfer 50 μl of each test solution to each well of the assay plate containing monolayers of these cells. The 485/535 emission ratio was calculated for each well and the mean ± SEM values for 12 wells were averaged. These FRET ratio values were normalized using baseline subtraction so that a y-axis value of 0 corresponds to the initial baseline FRET ratio, whereas a value of 100 corresponds to a 100% increase (*i.e.*, doubling) of the FRET ratio. The time course of the ΔFRET ratio was plotted after exporting data to Origin data analysis software (OriginLab). Origin was also used for nonlinear regression analysis to quantify dose-response relationships.

### Perifusion assays of rat islet GSIS

Sprague–Dawley rats obtained from Envigo RMS were fed a standard chow diet (TekLad Diet 2014; Harlan Laboratories) and were housed in an AAALC accredited vivarium at Lilly Research Laboratories. For 12-week-old male rats, the pancreas was surgically removed under conditions of isofluorane anesthesia after cervical dislocation, as stipulated in an animal use protocol approved by the Eli Lilly Institutional Animal Care and Use Committee. After inflation of the pancreas with a Hank’s balanced salt solution (Life Technologies, Cat. No. 14175–103) containing collagenase (VitaCyte, LCC; Cat. No. 005–1030), the pancreas was subjected to collagenase digestion (14 min) to obtain islets. These islets were cultured overnight in RPMI-1640 medium containing 11.1 mM glucose, 10% fetal bovine serum, glutamine (2 mM GlutaMax; Life Technologies), and penicillin-streptomycin. Perifusion assays of secreted insulin were performed the next day. Briefly, 50 islets were immobilized on a P-4 gel matrix (Bio-Gel, Bio-Rad Laboratories) within individual perifusion chambers housed in a 37 °C climate-controlled enclosure so that automated delivery of test solutions at a flow rate of 100 μl/min could be achieved using a Biorep Technologies Perifusion System. Thus, for each chamber, a peristaltic pump delivered Hepes-buffered saline solution containing (mM): 120 NaCl, 4.8 KCl, 2.5 CaCl_2_, 1.2 MgCl_2_, 10 Hepes, 24 Na_2_HCO_3_, and 0.25% BSA. The perifusate samples were collected at 4 °C using a robotic fraction collector (Biorep Tech.) designed for 96-well plates. Insulin content of the perifusates was quantified by electrochemical luminescence detection using an MSD Insulin Assay Kit (Meso Scale Diagnostics; Cat. No. K152BZC). The amount of secreted insulin present within each perifusate sample was normalized relative to islet DNA content for each chamber, as determined using a MagMax-96 DNA assay kit (Life Technologies; Cat. No. 4413021).

### Static incubation assays of mouse islet GSIS

C57BL/6J mice were obtained from Envigo RMS. Diet, housing, animal approval protocols, methods of islet isolation, and culture media were as described above for rats. Static incubation assays of GSIS were performed using freshly isolated islets after 2 h in culture or using islets cultured overnight for 24 h. Briefly, three islets were hand-picked into each well of a 48-well tissue culture plate containing 150 μl Hepes-buffered saline solution containing (mM): 120 NaCl, 4.8 KCl, 2.5 CaCl_2_, 1.2 MgCl_2_, 10 Hepes, 24 Na_2_HCO_3,_ 2.8 glucose, and 0.25% BSA. An additional 150 μl of solution was added so that the final concentration of glucose was either 2.8 or 11.2 mM without GLP-1 or with GLP-1 (6 wells/concentration tested). The plate containing islets was then incubated for 90 min at 37 °C in a cell culture incubator. The supernatant was collected, and insulin levels were quantified by electrochemical luminescence detection using an MSD insulin assay kit. The concentration-response relationships were established using GraphPad Prism software.

### Detection of glucagon and GLP-1

Detection of glucagon and GLP-1 in CM of rat islet and INS-1 832/13 cell cultures was performed after a 20 h exposure to culture medium. For this analysis, PIM(S) standard islet culture medium (Prodo Lab) was chosen because unlike RPMI-1640, it stabilizes glucagon so that repeated freeze-thawing is possible without the loss of GcgR stimulating properties. For each experiment, 300 rat islets were cultured in 4 ml of media, whereas for INS-1 832/13 cells, 4 ml was obtained from cultures at *ca.* 80% confluence. For detection of glucagon in the media, we used a Mercodia Glucagon ELISA-10 μl kit (Mercodia AB; Cat. No. 10-1281-01). For detection of total GLP-1, we used a Mercodia Total GLP-1 NL-ELISA kit (Cat. No. 10-1278-01) or an MSD V-PLEX kit (Meso Scale Diagnostics; Cat. No. K1503PD).

### Sources of reagents

Glucagon, LY2786890, and LY333531 were generated in-house at Lilly. GLP-1[7-36]amide (Cat. No. H-6795) and Ex[9–39] (Cat. No. H-8740) were from Bachem. des-His^1^-Glu^9^-Glucagon (Cat. No. 11084-95-2) was from Sigma Aldrich. U73122 (Cat. No. 112648-68-7), U73343 (Cat. No. 142878), and Ro 31-8220 were from Tocris. RPMI-1640 media (Cat. No. 61970-010) was from Gibco (Life Technologies). 6-Bnz-cAMP-AM (Cat. No. B 079) and 8-pCPT-2′-*O*-Me-cAMP-AM (Cat. No. C 051) were provided by Biolog Life Science Institute GmbH & Co KG. Rp-8-Br-cAMPS-pAB was provided by Biolog Life Science Institute for research purposes.

### Administration of test reagents to islets

At the start of each experiment, the GcgR, GLP-1R, and cAMP agonists or antagonists were preadministered to perifused islets as solutions in Hepes-buffered saline containing 2.8 mM glucose for 10 to 15 min. Such conditions favor α-cell glucagon release, while also suppressing β-cell insulin release (Fig. S6). Thus, at 2.8 mM glucose, intra-islet glucagon competes with administered GcgR and GLP-1R antagonists for binding to β-cell glucagon and GLP-1 receptors. These same test reagents were also present when islets were exposed to high concentrations of glucose, but they were absent upon reduction to 2.8 mM glucose at the end of each experiment.

### Statistical analyses

GSIS data presented in histogram format are the mean ± SEM. These data were evaluated for statistical significance by a one-way ANOVA test with Dunnett’s multiple comparisons test using GraphPad Prism v.9.1.2, which was also used to construct box and whiskers plots. Comparisons of individual data sets are defined in the accompanying figure legends. A *p* value of <0.05 was considered to be statistically significant. The box and whisker plots derived from these data show the following: mean (solid square), 25 to 75% range (open box), median (line across open box), and minimum and maximum values (whiskers). FRET data from individual experiments are expressed as the mean ± SEM and are derived from n = 12 wells for each concentration of test agent. The repeatability of findings was confirmed by performing each FRET experiment a minimum of three times.

## Data availability

The data that support the findings of this study are available from the corresponding authors upon reasonable request.

## Supporting information

This article contains supporting information.

## Conflict of interest

The authors declare that they have no conflicts of interest with the contents of this article.

## References

[1] Rorsman P., Braun M. (2013). Regulation of insulin secretion in human pancreatic islets. Annu. Rev. Physiol..

[2] Campbell J.E., Newgard C.B. (2021). Mechanisms controlling pancreatic islet cell function in insulin secretion. Nat. Rev. Mol. Cell Biol..

[3] Haeusler R.A., McGraw T.E., Accili D. (2018). Biochemical and cellular properties of insulin receptor signalling. Nat. Rev. Mol. Cell Biol..

[4] Galicia-Garcia U., Benito-Vicente A., Jebari S., Larrea-Sebal A., Siddiqi H., Uribe K.B., Ostolaza H., Martin C. (2020). Pathophysiology of type 2 diabetes mellitus. Int. J. Mol. Sci..

[5] Brunzell J.D., Robertson R.P., Lerner R.L., Hazzard W.R., Ensinck J.W., Bierman E.L., Porte D. (1976). Relationships between fasting plasma glucose levels and insulin secretion during intravenous glucose tolerance tests. J. Clin. Endocrinol. Metab..

[6] Gerich J.E. (2002). Is reduced first-phase insulin release the earliest detectable abnormality in individuals destined to develop type 2 diabetes?. Diabetes.

[7] Del Prato S., Marchetti P., Bonadonna R.C. (2002). Phasic insulin release and metabolic regulation in type 2 diabetes. Diabetes.

[8] Moens K., Heimberg H., Flamez D., Huypens P., Quartier E., Ling Z., Pipeleers D., Gremlich S., Thorens B., Schuit F. (1996). Expression and functional activity of glucagon, glucagon-like peptide I, and glucose-dependent insulinotropic peptide receptors in rat pancreatic islet cells. Diabetes.

[9] Moens K., Flamez D., Van Schravendijk C., Ling Z., Pipeleers D., Schuit F. (1998). Dual glucagon recognition by pancreatic beta-cells via glucagon and glucagon-like peptide 1 receptors. Diabetes.

[10] Huypens P., Ling Z., Pipeleers D., Schuit F. (2000). Glucagon receptors on human islet cells contribute to glucose competence of insulin release. Diabetologia.

[11] Moens K., Berger V., Ahn J.M., Van Schravendijk C., Hruby V.J., Pipeleers D., Schuit F. (2002). Assessment of the role of interstitial glucagon in the acute glucose secretory responsiveness of *in situ* pancreatic β-cells. Diabetes.

[12] Traub S., Meier D.T., Schulze F., Dror E., Nordmann T.M., Goetz N., Koch N., Dalmas E., Stawiski M., Makshana V., Thorel F., Herrera P.L., Boni-Schnetzler M., Donath M.Y. (2017). Pancreatic α cell-derived glucagon-related peptides are required for β cell adaptation and glucose homeostasis. Cell Rep..

[13] Rodriguez-Diaz R., Molano R.D., Weitz J.R., Abdulreda M.H., Berman D.M., Leibiger B., Leibiger I.B., Kenyon N.S., Ricordi C., Pileggi A., Caicedo A., Berggren P.O. (2018). Paracrine interactions within the pancreatic islet determine the glycemic set point. Cell Metab..

[14] Svendsen B., Larsen O., Gabe M.B.N., Christiansen C.B., Rosenkilde M.M., Drucker D.J., Holst J.J. (2018). Insulin secretion depends on intra-islet glucagon signaling. Cell Rep..

[15] Capozzi M.E., Svendsen B., Encisco S.E., Lewandowski S.L., Martin M.D., Lin H., Jaffe J.L., Coch R.W., Haldeman J.M., MacDonald P.E., Merrins M.J., D'Alessio D.A., Campbell J.E. (2019). β cell tone is defined by proglucagon peptides through cAMP signaling. JCI Insight.

[16] Zhu L., Dattaroy D., Pham J., Wang L., Barella L.F., Cui Y., Wilkins K.J., Roth B.L., Hochgeschwender U., Matschinsky F.M., Kaestner K.H., Doliba N.M., Wess J. (2019). Intra-islet glucagon signaling is critical for maintaining glucose homeostasis. JCI Insight.

[17] Gilon P. (2020). The role of α-cells in islet function and glucose homeostasis in health and type 2 diabetes. J. Mol. Biol..

[18] Moede T., Leibiger I.B., Berggren P.O. (2020). Alpha cell regulation of beta cell function. Diabetologia.

[19] Sandoval D. (2020). Updating the role of α-cell preproglucagon products on GLP-1 receptor-mediated insulin secretion. Diabetes.

[20] Henquin J.C. (2021). Paracrine and autocrine control of insulin secretion in human islets: Evidence and pending questions. Am. J. Physiol. Endocrinol. Metab..

[21] Nauck M., Weinstock R.S., Umpierrez G.E., Guerci B., Skrivanek Z., Milicevic Z. (2014). Efficacy and safety of dulaglutide versus sitagliptin after 52 weeks in type 2 diabetes in a randomized controlled trial (AWARD-5). Diabetes Care.

[22] Lau J., Bloch P., Schaffer L., Pettersson I., Spetzler J., Kofoed J., Madsen K., Knudsen L.B., McGuire J., Steensgaard D.B., Strauss H.M., Gram D.X., Knudsen S.M., Nielsen F.S., Thygesen P. (2015). Discovery of the once-weekly glucagon-like peptide-1 (GLP-1) analogue semaglutide. J. Med. Chem..

[23] Dungan K.M., Povedano S.T., Forst T., Gonzalez J.G., Atisso C., Sealls W., Fahrbach J.L. (2014). Once-weekly dulaglutide versus once-daily liraglutide in metformin-treated patients with type 2 diabetes (AWARD-6): A randomised, open-label, phase 3, non-inferiority trial. Lancet.

[24] Trujillo J.M., Nuffer W., Smith B.A. (2021). GLP-1 receptor agonists: An updated review of head-to-head clinical studies. Ther. Adv. Endocrinol. Metab..

[25] Schwede F., Chepurny O.G., Kaufholz M., Bertinetti D., Leech C.A., Cabrera O., Zhu Y., Mei F., Cheng X., Manning Fox J.E., MacDonald P.E., Genieser H.G., Herberg F.W., Holz G.G. (2015). Rp-cAMPS prodrugs reveal the cAMP dependence of first-phase glucose-stimulated insulin secretion. Mol. Endocrinol..

[26] Renstrom E., Eliasson L., Rorsman P. (1997). Protein kinase A-dependent and -independent stimulation of exocytosis by cAMP in mouse pancreatic β-cells. J. Physiol..

[27] Lester L.B., Langeberg L.K., Scott J.D. (1997). Anchoring of protein kinase A facilitates hormone-mediated insulin secretion. Proc. Natl. Acad. Sci. U. S. A..

[28] Hatakeyama H., Kishimoto T., Nemoto T., Kasai H., Takahashi N. (2006). Rapid glucose sensing by protein kinase A for insulin exocytosis in mouse pancreatic islets. J. Physiol..

[29] Seino S., Shibasaki T. (2005). PKA-dependent and PKA-independent pathways for cAMP-regulated exocytosis. Physiol. Rev..

[30] Song W.J., Seshadri M., Ashraf U., Mdluli T., Mondal P., Keil M., Azevedo M., Kirschner L.S., Stratakis C.A., Hussain M.A. (2011). Snapin mediates incretin action and augments glucose-dependent insulin secretion. Cell Metab..

[31] Henquin J.C., Nenquin M. (2014). Activators of PKA and Epac distinctly influence insulin secretion and cytosolic Ca^2+^ in female mouse islets stimulated by glucose and tolbutamide. Endocrinology.

[32] Holz G.G. (2004). Epac: A new cAMP-binding protein in support of glucagon-like peptide-1 receptor-mediated signal transduction in the pancreatic β-cell. Diabetes.

[33] Shibasaki T., Takahashi H., Miki T., Sunaga Y., Matsumura K., Yamanaka M., Zhang C., Tamamoto A., Satoh T., Miyazaki J., Seino S. (2007). Essential role of Epac2/Rap1 signaling in regulation of insulin granule dynamics by cAMP. Proc. Natl. Acad. Sci. U. S. A..

[34] Chepurny O.G., Leech C.A., Kelley G.G., Dzhura I., Dzhura E., Li X., Rindler M.J., Schwede F., Genieser H.G., Holz G.G. (2009). Enhanced Rap1 activation and insulin secretagogue properties of an acetoxymethyl ester of an Epac-selective cyclic AMP analog in rat INS-1 cells: Studies with 8-pCPT-2'-*O*-Me-cAMP-AM. J. Biol. Chem..

[35] Leech C.A., Chepurny O.G., Holz G.G. (2010). Epac2-dependent Rap1 activation and the control of islet insulin secretion by glucagon-like peptide-1. Vitam. Horm..

[36] Song W.J., Mondal P., Li Y., Lee S.E., Hussain M.A. (2013). Pancreatic β-cell response to increased metabolic demand and to pharmacologic secretagogues requires EPAC2A. Diabetes.

[37] Idevall-Hagren O., Jakobsson I., Xu Y., Tengholm A. (2013). Spatial control of Epac2 activity by cAMP and Ca^2+^-mediated activation of Ras in pancreatic β cells. Sci. Signal..

[38] Veluthakal R., Chepurny O.G., Leech C.A., Schwede F., Holz G.G., Thurmond D.C. (2018). Restoration of glucose-stimulated Cdc42-Pak1 activation and insulin secretion by a selective Epac activator in type 2 diabetic human islets. Diabetes.

[39] Holz G.G., Chepurny O.G., Schwede F. (2008). Epac-selective cAMP analogs: New tools with which to evaluate the signal transduction properties of cAMP-regulated guanine nucleotide exchange factors. Cell Signal..

[40] Jun L.S., Millican R.L., Hawkins E.D., Konkol D.L., Showalter A.D., Christe M.E., Michael M.D., Sloop K.W. (2015). Absence of glucagon and insulin action reveals a role for the GLP-1 receptor in endogenous glucose production. Diabetes.

[41] Chepurny O.G., Matsoukas M.T., Liapakis G., Leech C.A., Milliken B.T., Doyle R.P., Holz G.G. (2019). Nonconventional glucagon and GLP-1 receptor agonist and antagonist interplay at the GLP-1 receptor revealed in high-throughput FRET assays for cAMP. J. Biol. Chem..

[42] Schirra J., Sturm K., Leicht P., Arnold R., Goke B., Katschinski M. (1998). Exendin(9-39)amide is an antagonist of glucagon-like peptide-1(7-36)amide in humans. J. Clin. Invest..

[43] Serre V., Dolci W., Schaerer E., Scrocchi L., Drucker D., Efrat S., Thorens B. (1998). Exendin-(9-39) is an inverse agonist of the murine glucagon-like peptide-1 receptor: Implications for basal intracellular cyclic adenosine 3',5'-monophosphate levels and β-cell glucose competence. Endocrinology.

[44] Kofod H., Kirk O., Adelhorst K. (1996). β-Cell receptors for glucagon/GLP-1? Properties of exendin(9-39) in mouse islets. Acta Physiol. Scand..

[45] Bergman M., Abdul-Ghani M., DeFronzo R.A., Manco M., Sesti G., Fiorentino T.V., Ceriello A., Rhee M., Phillips L.S., Chung S., Cravalho C., Jagannathan R., Monnier L., Colette C., Owens D. (2020). Review of methods for detecting glycemic disorders. Diabetes Res. Clin. Pract..

[46] Hohmeier H.E., Mulder H., Chen G., Henkel-Rieger R., Prentki M., Newgard C.B. (2000). Isolation of INS-1-derived cell lines with robust ATP-sensitive K^+^ channel-dependent and -independent glucose-stimulated insulin secretion. Diabetes.

[47] Klarenbeek J., Goedhart J., van Batenburg A., Groenewald D., Jalink K. (2015). Fourth-generation Epac-based FRET sensors for cAMP feature exceptional brightness, photostability and dynamic range: Characterization of dedicated sensors for FLIM, for ratiometry and with high affinity. PLoS One.

[48] Allen M.D., Zhang J. (2006). Subcellular dynamics of protein kinase A activity visualized by FRET-based reporters. Biochem. Biophys. Res. Commun..

[49] Shigeto M., Katsura M., Matsuda M., Ohkuma S., Kaku K. (2008). Low, but physiological, concentration of GLP-1 stimulates insulin secretion independent of the cAMP-dependent protein kinase pathway. J. Pharmacol. Sci..

[50] Shigeto M., Ramracheya R., Tarasov A.I., Cha C.Y., Chibalina M.V., Hastoy B., Philippaert K., Reinbothe T., Rorsman N., Salehi A., Sones W.R., Vergari E., Weston C., Gorelik J., Katsura M. (2015). GLP-1 stimulates insulin secretion by PKC-dependent TRPM4 and TRPM5 activation. J. Clin. Invest..

[51] Shigeto M., Cha C.Y., Rorsman P., Kaku K. (2017). A role of PLC/PKC-dependent pathway in GLP-1-stimulated insulin secretion. J. Mol. Med. (Berl.).

[52] Kelley G.G., Chepurny O.G., Schwede F., Genieser H.G., Leech C.A., Roe M.W., Li X., Dzhura I., Dzhura E., Afshari P., Holz G.G. (2009). Glucose-dependent potentiation of mouse islet insulin secretion by Epac activator 8-pCPT-2'-*O*-Me-cAMP-AM. Islets.

[53] Dzhura I., Chepurny O.G., Leech C.A., Roe M.W., Dzhura E., Xu X., Lu Y., Schwede F., Genieser H.G., Smrcka A.V., Holz G.G. (2011). Phospholipase C-ε links Epac2 activation to the potentiation of glucose-stimulated insulin secretion from mouse islets of Langerhans. Islets.

[54] Dzhura I., Chepurny O.G., Kelley G.G., Leech C.A., Roe M.W., Dzhura E., Afshari P., Malik S., Rindler M.J., Xu X., Lu Y., Smrcka A.V., Holz G.G. (2010). Epac2-dependent mobilization of intracellular Ca^2+^ by glucagon-like peptide-1 receptor agonist exendin-4 is disrupted in β-cells of phospholipase C-ε knockout mice. J. Physiol..

[55] Henquin J.C. (2021). Glucose-induced insulin secretion in isolated human islets: Does it truly reflect β-cell function *in vivo*?. Mol. Metab..

[56] Pipeleers D., in't Veld P.I., Maes E., Van De Winkel M. (1982). Glucose-induced insulin release depends on functional cooperation between islet cells. Proc. Natl. Acad. Sci. U. S. A..

[57] Pipeleers D.G., Schuit F.C., in't Veld P.A., Maes E., Hooghe-Peters E.L., Van de Winkel M., Gepts W. (1985). Interplay of nutrients and hormones in the regulation of insulin release. Endocrinology.

[58] Schuit F.C., Pipeleers D.G. (1985). Regulation of adenosine 3',5'-monophosphate levels in the pancreatic B cell. Endocrinology.

[59] Holz G.G., Habener J.F. (1992). Signal transduction crosstalk in the endocrine system: Pancreatic β-cells and the glucose competence concept. Trends. Biochem. Sci..

[60] Holz G.G., Kuhtreiber W.M., Habener J.F. (1993). Pancreatic beta-cells are rendered glucose-competent by the insulinotropic hormone glucagon-like peptide-1(7-37). Nature.

[61] Mojsov S., Heinrich G., Wilson I.B., Ravazzola M., Orci L., Habener J.F. (1986). Preproglucagon gene expression in pancreas and intestine diversifies at the level of post-translational processing. J. Biol. Chem..

[62] Whalley N.M., Pritchard L.E., Smith D.M., White A. (2011). Processing of proglucagon to GLP-1 in pancreatic α-cells: Is this a paracrine mechanism enabling GLP-1 to act on β-cells?. J. Endocrinol..

[63] O'Malley T.J., Fava G.E., Zhang Y., Fonseca V.A., Wu H. (2014). Progressive change of intra-islet GLP-1 production during diabetes development. Diabetes Metab. Res. Rev..

[64] Marchetti P., Lupi R., Bugliani M., Kirkpatrick C.L., Sebastiani G., Grieco F.A., Del Guerra S., D'Aleo V., Piro S., Marselli L., Boggi U., Filipponi F., Tinti L., Salvini L., Wollheim C.B. (2012). A local glucagon-like peptide 1 (GLP-1) system in human pancreatic islets. Diabetologia.

[65] Campbell S.A., Golec D.P., Hubert M., Johnson J., Salamon N., Barr A., MacDonald P.E., Philippaert K., Light P.E. (2020). Human islets contain a subpopulation of glucagon-like peptide-1 secreting α cells that is increased in type 2 diabetes. Mol. Metab..

[66] Wideman R.D., Covey S.D., Webb G.C., Drucker D.J., Kieffer T.J. (2007). A switch from prohormone convertase (PC)-2 to PC1/3 expression in transplanted α-cells is accompanied by differential processing of proglucagon and improved glucose homeostasis in mice. Diabetes.

[67] Holst J.J., Bersani M., Johnsen A.H., Kofod H., Hartmann B., Orskov C. (1994). Proglucagon processing in porcine and human pancreas. J. Biol. Chem..

[68] Galvin S.G., Kay R.G., Foreman R., Larraufie P., Meek C.L., Biggs E., Ravn P., Jermutus L., Reimann F., Gribble F.M. (2021). The human and mouse islet peptidome: Effects of obesity and type 2 diabetes, and assessment of intraislet production of glucagon-like peptide-1. J. Proteome Res..

[69] Kuhtreiber W.M., Ho L.T., Kamireddy A., Yacoub J.A., Scharp D.W. (2010). Islet isolation from human pancreas with extended cold ischemia time. Transpl. Proc..

[70] Zaccolo M., Zerio A., Lobo M.J. (2021). Subcellular organization of the cAMP signaling pathway. Pharmacol. Rev..

[71] Henquin J.C., Nenquin M., Stiernet P., Ahren B. (2006). *In vivo* and *in vitro* glucose-induced biphasic insulin secretion in the mouse: Pattern and role of cytoplasmic Ca^2+^ and amplification signals in beta-cells. Diabetes.

[72] Gelling R.W., Du X.Q., Dichmann D.S., Romer J., Huang H., Cuim L., Obicim S., Tang B., Holst J.J., Fledelius C., Johansen P.B., Rossetti L., Jelicks L.A., Serup P., Nishimura E. (2003). Lower blood glucose, hyperglucagonemia, and pancreatic alpha cell hyperplasia in glucagon receptor knockout mice. Proc. Natl. Acad. Sci. U. S. A..

[73] Sørensen H., Winzell S., Brand C.L., Fosgerau K., Gelling R.W., Nishimura E., Ahren B. (2006). Glucagon receptor knockout mice display increased insulin sensitivity and impaired β-cell function. Diabetes.

[74] Zhang Y., Han C., Zhu W., Yang G., Peng X., Mehta S., Zhang J., Chen L., Liu Y. (2021). Glucagon potentiates insulin secretion via β-cell GCGR at physiological concentrations of glucose. Cells.

[75] Shuai H., Xu Y., Ahooghalandari P., Tengholm A. (2021). Glucose-induced cAMP elevation in β-cells involves amplification of constitutive and glucagon-activated GLP-1 receptor signalling. Acta Physiol. (Oxf.).

[76] Fehse F., Trautmann M., Holst J.J., Halseth A.E., Nanayakkara N., Nielsen L.L., Fineman M.S., Kim D.D., Nauck M.A. (2005). Exenatide augments first- and second-phase insulin secretion in response to intravenous glucose in subjects with type 2 diabetes. J. Clin. Endocrinol. Metab..

[77] Finan B., Capozzi M.E., Campbell J.E. (2020). Repositioning glucagon action in the physiology and pharmacology of diabetes. Diabetes.

[78] Müller T.D., Finan B., Clemmensen C., DiMarchi R.D., Tschöp M.H. (2017). The new biology and pharmacology of glucagon. Physiol. Rev..

[79] Jiang Y., Cypess A.M., Muse E.D., Wu C.R., Unson C.G., Merrifield R.B., Sakmar T.P. (2001). Glucagon receptor activates extracellular signal-regulated protein kinase 1/2 via cAMP-dependent protein kinase. Proc. Natl. Acad. Sci. U. S. A..

[80] Gromada J., Rorsman P., Dissing S., Wulff B.S. (1995). Stimulation of cloned human glucagon-like peptide 1 receptor expressed in HEK 293 cells induces cAMP-dependent activation of calcium-induced calcium release. FEBS Lett..

[81] Chepurny O.G., Bonaccorso R.L., Leech C.A., Wollert T., Langford G.M., Schwede F., Roth C.L., Doyle R.P., Holz G.G. (2018). Chimeric peptide EP45 as a dual agonist at GLP-1 and NPY2R receptors. Sci. Rep..

[82] Tibaduiza E.C., Chen C., Beinborn M. (2001). A small molecule ligand of the glucagon-like peptide 1 receptor targets its amino-terminal hormone binding domain. J. Biol. Chem..

