# Supplementary material for: Intra-islet glucagon confers β-cell glucose competence for first-phase insulin secretion and favors GLP-1R stimulation by exogenous glucagon
Source: J Biol Chem. 2021 Dec 9;298(2):101484. doi: 10.1016/j.jbc.2021.101484 (PMC8789663; doi:10.1016/j.jbc.2021.101484)
Supplement: Supplemental Figures S1–S6 [file mmc1.pdf]

Supporting Information

Intra-islet glucagon confers β-cell glucose competence for first-phase insulin secretion and favors GLP-1R stimulation by exogenous glucagon

Over Cabrera[*1], James Ficorilli[1], Janice Shaw[1], Felipe Echeverri[2], Frank Schwede[3], Oleg G. Chepurny[4], Colin A. Leech[4], George G. Holz[*4,5]

[1]Lilly Research Laboratories, Eli Lilly and Company, 307 East Merrill St., Indianapolis, IN 46225 (USA)

[2]Biorep Technologies, 15804 NW 57th Avenue, Miami Lakes, FL 33014 (USA)

[3]Biolog Life Science Institute GmbH & Co. KG Flughafendamm 9a, 28199 Bremen, Germany

Departments of Medicine[4] and Pharmacology[5]
State University of New York (SUNY) Upstate Medical University
505 Irving Avenue, Syracuse, NY 13210 (USA)

Running Title: *Glucagon regulation of rat islet insulin secretion*

[*]To whom correspondence should be addressed:
Over G. Cabrera (e-mail: over.cabrera@lilly.com; Telephone 317-443-9593)
George G. Holz (e-mail: holzg@upstate.edu; Telephone 315-464-9841)

Key words: Glucagon, GLP-1, cAMP, islet, insulin secretion

Supporting Information Consists Of The Following:

Figures and Legends for S1 – S6

Figure Legends

Figure S1. Extremely low concentrations of GLP-1 fail to potentiate mouse islet GSIS. (*a,b*) Illustrated are findings from static incubation assays of insulin secretion using 24 hr. cultured mouse islets equilibrated in buffer containing either 6 mM (*a*) or 12 mM (*b*) glucose. Note that the secretagogue action of GLP-1 to potentiate GSIS was only significant when testing 10 or 30 nM of the peptide. (*c,d*) Illustrated are GLP-1 concentration-response relationships for static incubation assays performed using 24 hr. cultured islets (*c*), or islets that were tested immediately after harvesting (*d*). For all panels, data are replicates of 4 independent batches of islets per concentration of GLP-1. Error bars indicate mean +/- s.e.m. Statistical significance was evaluated by one-way ANOVA followed by Dunnett's post-hoc test to compare GLP-1 action at 6 or 12 mM glucose relative to islets not receiving GLP-1.

Figure S2. Pharmacological properties of PLC and PKC inhibitors tested in assays of rat islet GSIS. (a_1,a_2) Pretreatment for 40 min with the PLC inhibitor U73122 (1 μM), and the negative control U73343 (1 μM) that does not inhibit PLC, failed to block the potentiation of GSIS by GLP-1 (1 nM). (b_1,b_2) PKC inhibitors LY 333531 (10 μM) and Ro 31-8220 (10 μM) also failed to block the potentiation of GSIS by GLP-1.

Figure S3. Potentiation of 1st and 2nd phase GSIS by GLP-1 is unaffected by the GRA. (a_1) GLP-1 (1 nM) potentiated GSIS, and this action of GLP-1 was unaffected by treatment of islets with the GRA (70 nM). (a_2) Expanded time base derived from panel a_1 demonstrating that 1st phase GSIS potentiated by GLP-1 was unaffected by the GRA. Data for both panels are averages of the same 3 independent experiments.

Figure S4. GcgR antagonist des-His^{1}-Glu^{9}-glucagon fails to inhibit GSIS stimulated by a linear gradient of increasing glucose concentrations.

(a_1-a_3) des-His^{1}-Glu^{9}-glucagon (3 μM) failed to inhibit GSIS in response to glucose alone ($a_{1,3}$). Glucagon (10 nM) potentiated GSIS, an effect not inhibited by des-His^{1}-Glu^{9}-glucagon ($a_{2,3}$). Results were obtained in a single experiment. Panel a_3 illustrates box and whiskers AUC analysis with accompanying ANOVA derived P values where each symbol is the AUC value for islets of a single perifusion chamber. Islets were perifused under conditions in which the buffer's initial glucose concentration was 3 mM, after which the glucose concentration increased at a rate of 0.27 mM/min, starting at 3.3 mM at t=15 min, and ending at 30.3 mM at t= 65 min, after which the glucose concentration was stepped down to 3.0 mM.

Figure S5. GLP-1 acts at the GLP-1R but not GcgR to potentiate GSIS stimulated by a linear gradient of increasing glucose concentrations.

(a_1,a_2) GLP-1 (10 nM) potentiated GSIS in the glucose gradient assay, and this action of GLP-1 was not inhibited by the GRA. (b_1,b_2) The action of GLP-1 (10 nM) to potentiate GSIS in the gradient assay was fully blocked by Ex[9-39] (1 μM). Findings illustrated in panels $a_{1,2}$ and $b_{1,2}$ are from single independent experiments. Panesl a_2 *and* b_2 illustrate box and whiskers AUC analysis with accompanying ANOVA derived P values where each symbol is the AUC value for islets of a single perifusion chamber. Islets were perifused under conditions in which the buffer's initial glucose concentration was 3 mM, after which the glucose concentration increased at a rate of 0.27 mM/min, starting at 3.3 mM at t=15 min, and ending at 30.3 mM at t= 65 min, after which the glucose concentration was stepped down to 3.0 mM.

Figure S6. Reciprocal changes of glucagon and insulin levels measured in perifusates of rat islets after stepping from 2.8 to 16.7 mM glucose.

Preequilibration of islets in 2.8 mM glucose resulted in α-cell glucagon secretion, and under these conditions the perifusate's glucagon concentration was 5.9 +/- 0.5 pM. Stepping the glucose concentration to 16.7 mM led to a rapid suppression of glucagon secretion so that levels of glucagon were reduced to the limit of immunodetection. Note that for these same islets, β-cell insulin secretion was stimulated at 16.7 mM glucose thereby generating 1st and 2nd phase GSIS. Findings are averaged data from one experiment using 3 rats and 12 pooled batches of islets in which all perifusates were collected simultaneously on a single day. Data are presented as the mean +/- s.e.m for individual time points.

Fig.S1

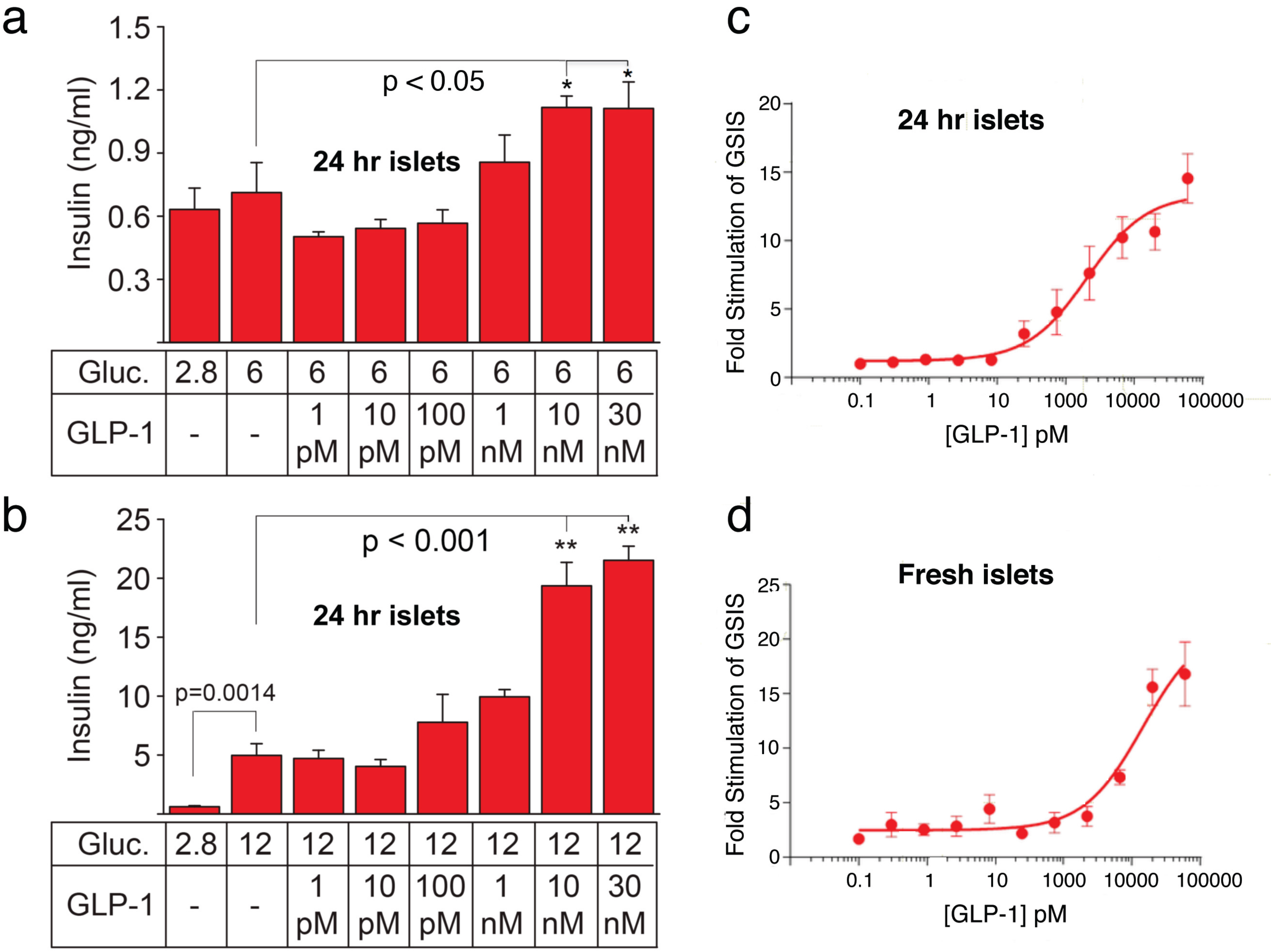

a
Insulin (ng/ml)
1.5
1.2
0.9
0.6
0.3
p < 0.05
24 hr islets
*
*
Gluc. 2.8 6 6 6 6 6 6 6
GLP-1 - - 1 pM 10 pM 100 pM 1 nM 10 nM 30 nM
b
Insulin (ng/ml)
25
20
15
10
5
p < 0.001
24 hr islets
**
**
p=0.0014
Gluc. 2.8 12 12 12 12 12 12 12
GLP-1 - - 1 pM 10 pM 100 pM 1 nM 10 nM 30 nM
c
24 hr islets
Fold Stimulation of GSIS
20
15
10
5
0
0.1
1
10
1000
10000
100000
[GLP-1] pM
d
Fresh islets
Fold Stimulation of GSIS
25
20
15
10
5
0
0.1
1
10
1000
10000
100000
[GLP-1] pM

Fig.S2

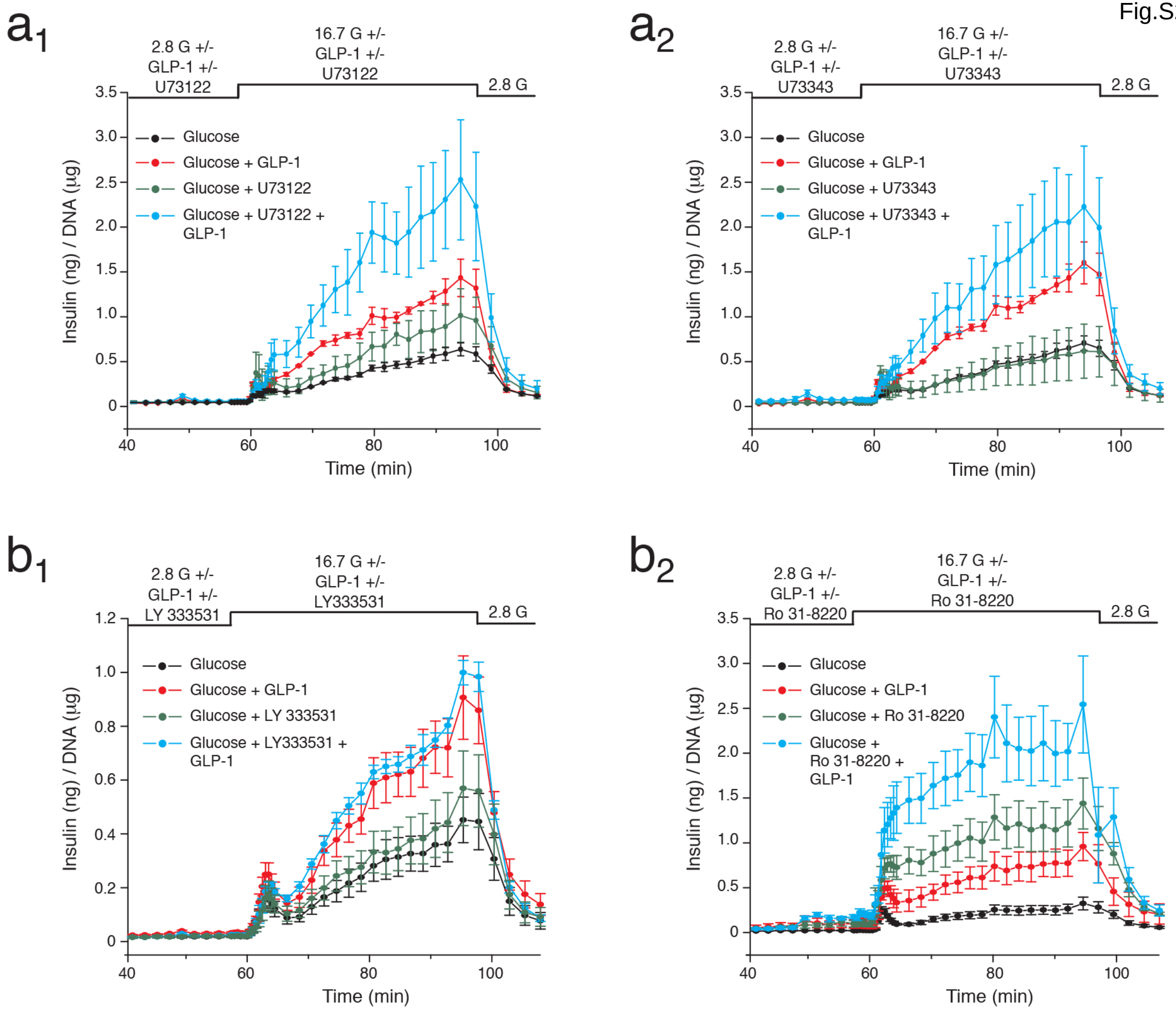

a1
2.8 G +/- GLP-1 +/- U73122
16.7 G +/- GLP-1 +/- U73122
2.8 G
Glucose
Glucose + GLP-1
Glucose + U73122
Glucose + U73122 + GLP-1
Insulin (ng) / DNA (µg)
Time (min)
a2
2.8 G +/- GLP-1 +/- U73343
16.7 G +/- GLP-1 +/- U73343
2.8 G
Glucose
Glucose + GLP-1
Glucose + U73343
Glucose + U73343 + GLP-1
Insulin (ng) / DNA (µg)
Time (min)
b1
2.8 G +/- GLP-1 +/- LY 333531
16.7 G +/- GLP-1 +/- LY333531
2.8 G
Glucose
Glucose + GLP-1
Glucose + LY 333531
Glucose + LY333531 + GLP-1
Insulin (ng) / DNA (µg)
Time (min)
b2
2.8 G +/- GLP-1 +/- Ro 31-8220
16.7 G +/- GLP-1 +/- Ro 31-8220
2.8 G
Glucose
Glucose + GLP-1
Glucose + Ro 31-8220
Glucose + Ro 31-8220 + GLP-1
Insulin (ng) / DNA (µg)
Time (min)

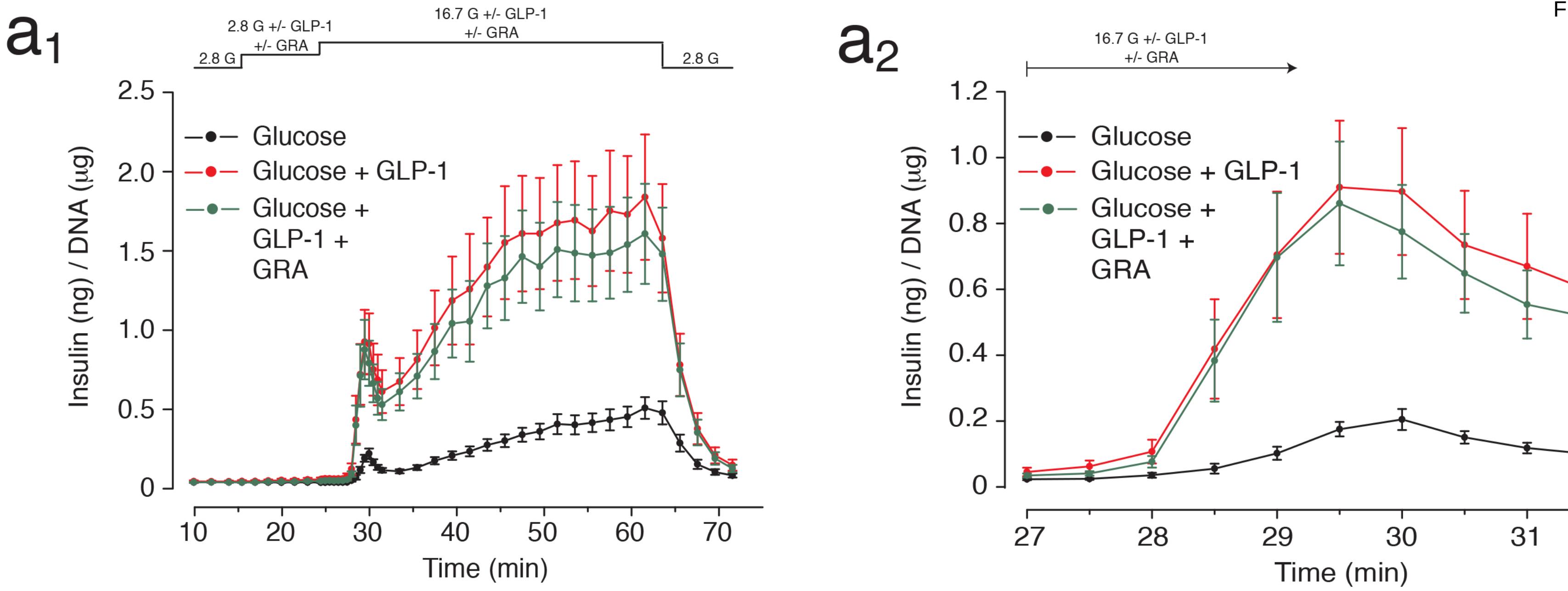
a1
2.8 G
2.8 G +/- GLP-1
+/- GRA
16.7 G +/- GLP-1
+/- GRA
2.8 G
Glucose
Glucose + GLP-1
Glucose +
GLP-1 +
GRA
Insulin (ng) / DNA (µg)
Time (min)
0
0.5
1.0
1.5
2.0
2.5
10
20
30
40
50
60
70
a2
16.7 G +/- GLP-1
+/- GRA
Glucose
Glucose + GLP-1
Glucose +
GLP-1 +
GRA
Insulin (ng) / DNA (µg)
Time (min)
0
0.2
0.4
0.6
0.8
1.0
1.2
27
28
29
30
31

Fig.S4

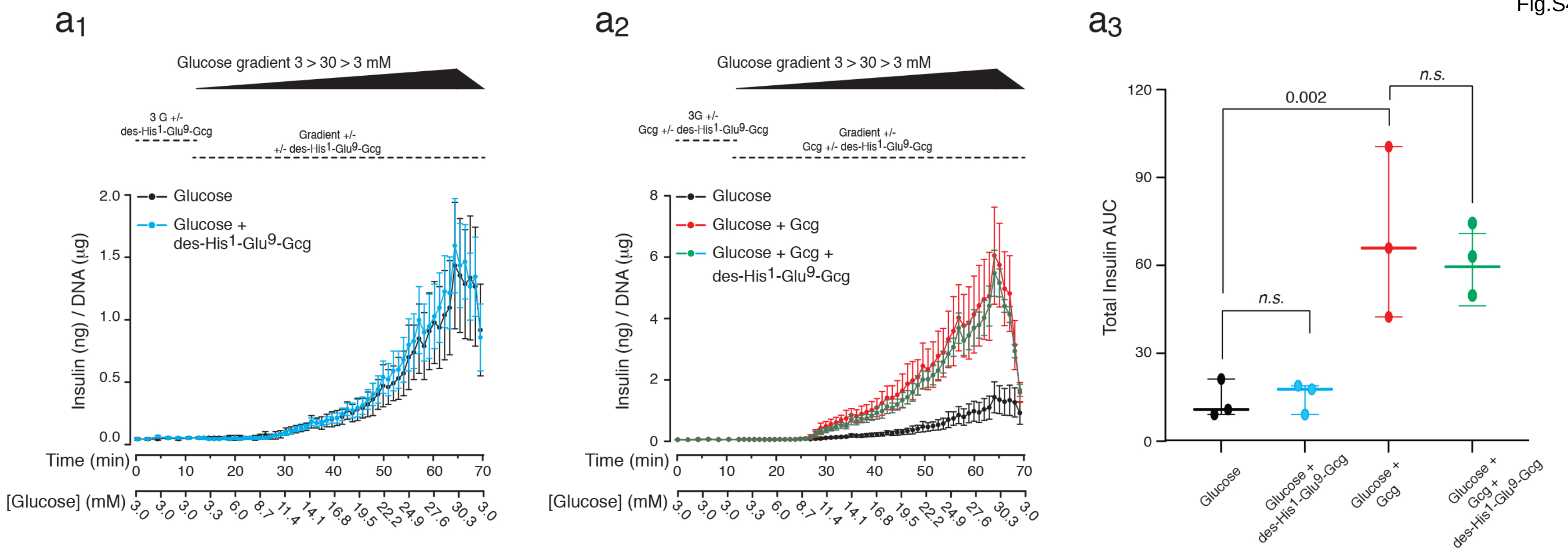
a1
Glucose gradient 3 > 30 > 3 mM
3 G +/- des-His1-Glu9-Gcg
Gradient +/- +/- des-His1-Glu9-Gcg
Glucose
Glucose + des-His1-Glu9-Gcg
Insulin (ng) / DNA (µg)
Time (min)
[Glucose] (mM)
a2
Glucose gradient 3 > 30 > 3 mM
3G +/- Gcg +/- des-His1-Glu9-Gcg
Gradient +/- Gcg +/- des-His1-Glu9-Gcg
Glucose
Glucose + Gcg
Glucose + Gcg + des-His1-Glu9-Gcg
Insulin (ng) / DNA (µg)
Time (min)
[Glucose] (mM)
a3
Total Insulin AUC
0.002
n.s.
n.s.
Glucose
Glucose + des-His1-Glu9-Gcg
Glucose + Gcg
Glucose + Gcg + des-His1-Glu9-Gcg

Fig.S5

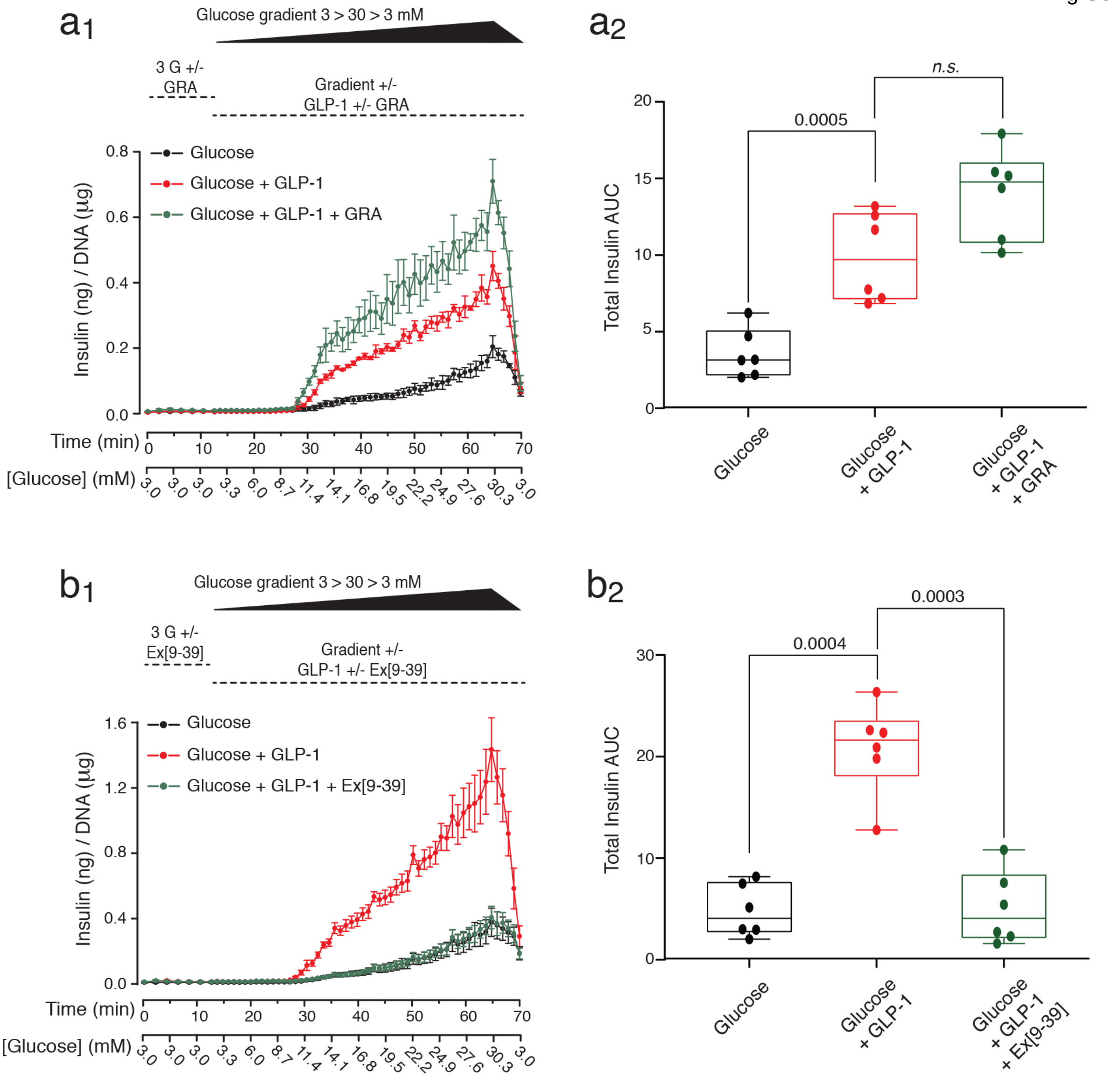
a1
Glucose gradient 3 > 30 > 3 mM
3 G +/- GRA
Gradient +/- GLP-1 +/- GRA
Glucose
Glucose + GLP-1
Glucose + GLP-1 + GRA
Insulin (ng) / DNA (µg)
0.0
0.2
0.4
0.6
0.8
Time (min)
0
10
20
30
40
50
60
70
[Glucose] (mM)
3.0
3.0
3.0
3.3
6.0
8.7
11.4
14.1
16.8
19.5
22.2
24.9
27.6
30.3
3.0
a2
Total Insulin AUC
0
5
10
15
20
0.0005
n.s.
Glucose
Glucose + GLP-1
Glucose + GLP-1 + GRA
b1
Glucose gradient 3 > 30 > 3 mM
3 G +/- Ex[9-39]
Gradient +/- GLP-1 +/- Ex[9-39]
Glucose
Glucose + GLP-1
Glucose + GLP-1 + Ex[9-39]
Insulin (ng) / DNA (µg)
0.0
0.4
0.8
1.2
1.6
Time (min)
0
10
20
30
40
50
60
70
[Glucose] (mM)
3.0
3.0
3.0
3.3
6.0
8.7
11.4
14.1
16.8
19.5
22.2
24.9
27.6
30.3
3.0
b2
Total Insulin AUC
0
10
20
30
0.0004
0.0003
Glucose
Glucose + GLP-1
Glucose + GLP-1 + Ex[9-39]

Fig.S6

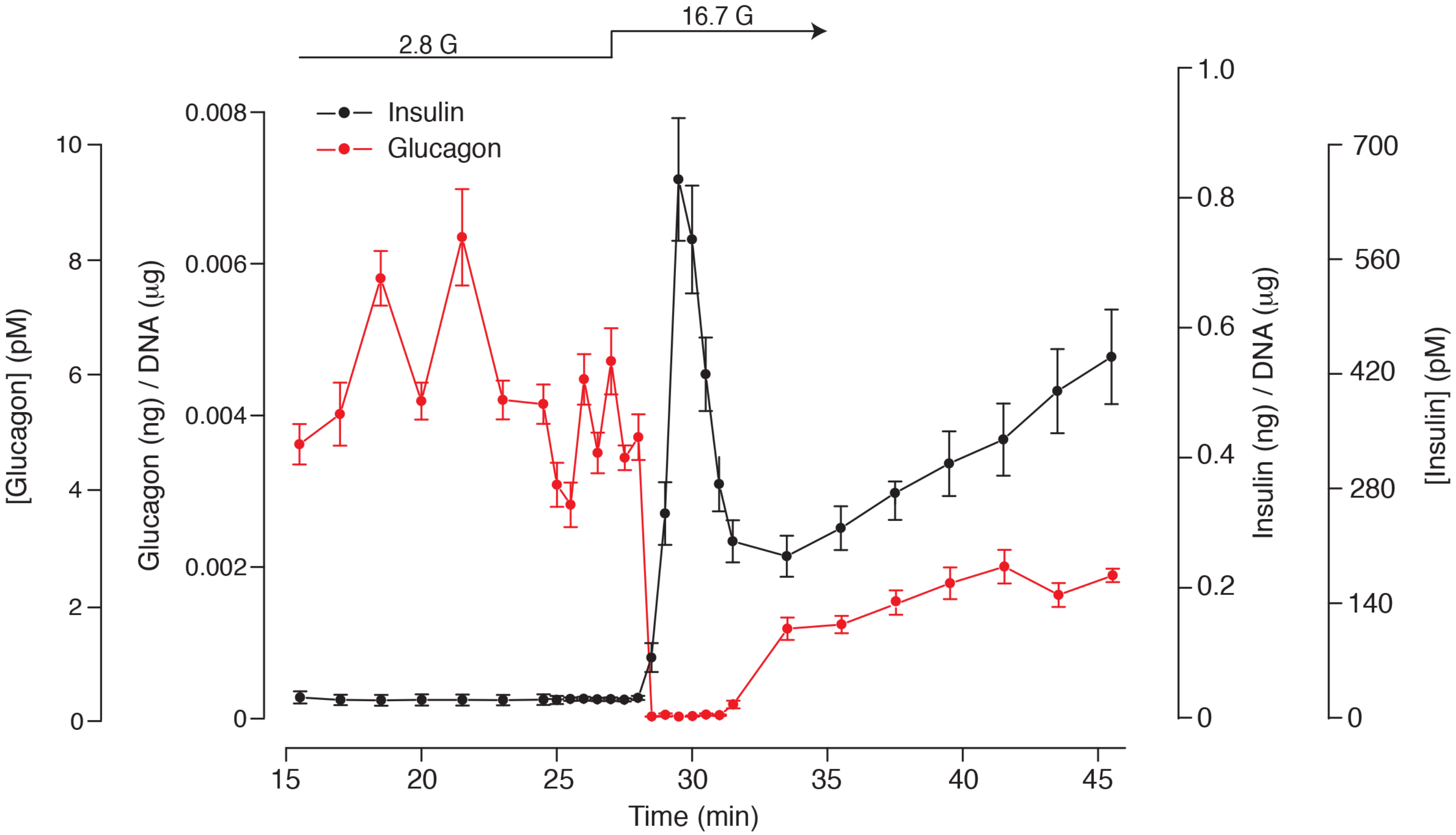

2.8 G
16.7 G
Insulin
Glucagon
[Glucagon] (pM)
0
2
4
6
8
10
Glucagon (ng) / DNA (µg)
0
0.002
0.004
0.006
0.008
Insulin (ng) / DNA (µg)
0
0.2
0.4
0.6
0.8
1.0
[Insulin] (pM)
0
140
280
420
560
700
Time (min)
15
20
25
30
35
40
45
